# eIF3 and Its mRNA-Entry-Channel Arm Contribute to the Recruitment of mRNAs With Long 5′-Untranslated Regions

**DOI:** 10.3389/fmolb.2021.787664

**Published:** 2022-01-11

**Authors:** Andrei Stanciu, Juncheng Luo, Lucy Funes, Shanya Galbokke Hewage, Shardul D. Kulkarni, Colin Echeverría Aitken

**Affiliations:** ^1^ Computer Science Department, Vassar College, Poughkeepsie, NY, United States; ^2^ Biochemistry Program, Vassar College, Poughkeepsie, NY, United States; ^3^ Biology Department, Vassar College, Poughkeepsie, NY, United States; ^4^ Department of Biochemistry and Molecular Biology, Penn State Eberly College of Medicine, University Park, PA, United States

**Keywords:** eIF3, translation initiation, translational regulation, mRNA recruitment, ribosome, ribosome profiling, ribo-seq

## Abstract

Translation initiation in eukaryotes is a multi-step pathway and the most regulated phase of translation. Eukaryotic initiation factor 3 (eIF3) is the largest and most complex of the translation initiation factors, and it contributes to events throughout the initiation pathway. In particular, eIF3 appears to play critical roles in mRNA recruitment. More recently, eIF3 has been implicated in driving the selective translation of specific classes of mRNAs. However, unraveling the mechanism of these diverse contributions—and disentangling the roles of the individual subunits of the eIF3 complex—remains challenging. We employed ribosome profiling of budding yeast cells expressing two distinct mutations targeting the eIF3 complex. These mutations either disrupt the entire complex or subunits positioned near the mRNA-entry channel of the ribosome and which appear to relocate during or in response to mRNA binding and start-codon recognition. Disruption of either the entire eIF3 complex or specific targeting of these subunits affects mRNAs with long 5′-untranslated regions and whose translation is more dependent on eIF4A, eIF4B, and Ded1 but less dependent on eIF4G, eIF4E, and PABP. Disruption of the entire eIF3 complex further affects mRNAs involved in mitochondrial processes and with structured 5′-untranslated regions. Comparison of the suite of mRNAs most sensitive to both mutations with those uniquely sensitive to disruption of the entire complex sheds new light on the specific roles of individual subunits of the eIF3 complex.

## Introduction

Translation initiation is the rate-limiting and most regulated phase of translation (Sonenberg and Hinnebusch, 2009; Jackson et al., 2010). Translation initiation in eukaryotes requires the ribosome—the macromolecular machine responsible for synthesizing the proteins encoded by messenger RNA molecules in all kingdoms of life—to dock at the very 5′ end of a mRNA molecule and then scan to identify the start codon for translation, usually the first AUG. The sequence through which the ribosome must scan, known either as the 5′-untranslated region (5′-UTR) or the transcript leader (TL), can be in excess of a 1,000 nucleotides in length and contain regions of defined secondary structure or upstream open reading frames (uORFS) demarcated by either cognate (AUG) or near-cognate start codons and whose translation can regulate translation of the downstream open reading frame (ORF) (Hinnebusch et al., 2016).

At least twelve protein initiation factors (eIFs) collaborate with the ribosome to facilitate its navigation of these obstacles (Aitken and Lorsch, 2012; Shivaya Valasek, 2012; Hinnebusch, 2014; Hinnebusch, 2017). The process begins with the formation of a pre-initiation complex (PIC) comprising the small (40S) ribosomal subunit, a ternary complex (TC) of the initiator methionyl tRNA (tRNA_i_), the GTPase eIF2, and GTP (tRNA_i_•eIF2•GTP), and the initiation factors eIF1, eIF1A, eIF5, and eIF3. The PIC then docks at the 5′ end of the mRNA in collaboration with the eIF4F complex comprising the cap-binding protein eIF4E, the scaffolding protein eIF4G, and the helicase eIF4A, which may facilitate initial docking of the PIC by relaxing structural complexity near the 5′ end of the mRNA (Yourik et al., 2017). Once docked at the 5′ end, the PIC scans in the 3′ direction to identify the start codon. Scanning is thought to be facilitated by eIF4A and eIF4B, which binds the 40S subunit (Walker et al., 2013). The helicase Ded1 also plays an important role in scanning, perhaps by resolving defined structural elements within the 5’ UTR that might otherwise prevent efficient scanning (Gupta et al., 2018).

The largest and most complex of the initiation factors is eIF3, a multi-subunit complex comprising at least 5 essential subunits in the yeast *S. cerevisiae* and at least 12 subunits in mammalian cells (Hinnebusch, 2006; Valášek et al., 2017). eIF3 participates in every component step of translation initiation. It stabilizes and promotes formation of the PIC via interactions with the 40S subunit, eIF1, and eIF2 within the TC (Asano et al., 2000; Valášek et al., 2002; Majumdar et al., 2003; Valášek et al., 2003; Nielsen et al., 2006; Sokabe and Fraser, 2014). eIF3 is also required, both *in vivo* and *in vitro*, for mRNA recruitment by the PIC (Jivotovskaya et al., 2006; Mitchell et al., 2010; Aitken et al., 2016), a process consisting of PIC docking, scanning, and start-codon recognition. Consistent with this role, eIF3 binds the PIC at the solvent face but projects appendages near both the mRNA-entry and mRNA-exit channels of the ribosome (Aylett et al., 2015; Des Georges et al., 2015; Llácer et al., 2018). At the mRNA-exit channel, the eIF3a subunit (and eIF3d in higher eukaryotes) appears to interact functionally or physically with the mRNA (Szamecz et al., 2008; Munzarová et al., 2011) and the very N-terminal region of eIF3a seems to stabilize the binding of mRNA to the PIC (Aitken et al., 2016). Subunits of the human eIF3 complex bind to eIF4G (Villa et al., 2013), and some of these were found interacting directly with components of the eIF4F complex at the mRNA-exit channel in a recent high-resolution structure of the human 48S PIC (Querido et al., 2020).

Near the mRNA-entry channel, the C-terminal domain (CTD) of eIF3a interacts with 40S elements that mediate the transition between the open (docking- and scanning-competent) and closed (scanning-arresting) conformations of the PIC (Chiu et al., 2010; Dong et al., 2017). High-resolution structural models of eIF3 bound to the PIC reveal that the eIF3a CTD, eIF3b, eIF3i, and eIF3g compose this mRNA-entry-channel arm (Des Georges et al., 2015; Simonetti et al., 2016; Llácer et al., 2018). Moreover, structural models of the PIC either lacking or bound to mRNA reveal distinct positions of this arm (Llácer et al., 2015; Llácer et al., 2018). In the absence of mRNA, these subunits are found bound to the solvent face of the PIC; in the presence of mRNA, but prior to start-codon recognition, the mRNA-entry-channel arm is found at the intersubunit face but then appears to relocate to its original position at the solvent face upon start-codon recognition. Together with the observations that mutations to the eIF3a CTD (Valášek et al., 2002; Chiu et al., 2010), eIF3b (Nielsen et al., 2004; Elantak et al., 2010), eIF3i (Herrmannová et al., 2012), and eIF3g (Cuchalova et al., 2010) elicit phenotypes consistent with defects in the component events of mRNA recruitment and affect the kinetics of mRNA recruitment *in vitro* (Aitken et al., 2016), this suggests that the eIF3 mRNA-entry-channel arm, and its potential repositioning in response to mRNA binding and start-codon recognition, may play an important mechanistic role in mRNA recruitment.

To investigate the role of eIF3 and components of the eIF3 mRNA-entry-channel arm in mRNA recruitment and its component events, we employed ribosome profiling (Ingolia et al., 2009) to follow the repercussions of specific eIF3 mutations on the translational efficiency (TE) of mRNAs across the transcriptome. By comparing the features of mRNAs most sensitive to each mutation with those least sensitive to these mutations, we shed new light on the role of eIF3 and its mRNA-entry-channel arm in mRNA recruitment. This approach has previously been employed to illuminate the transcriptome-scale role of several initiation factors, including eIF1 (Zhou et al., 2020), eIF1A (Martin-Marcos et al., 2017), eIF4A (Sen et al., 2015), eIF4B (Sen et al., 2016), and Ded1 (Sen et al., 2019). Here, we focused on two mutations whose effects on translation initiation have been previously explored with both genetic and biochemical tools. The first of these mutants—*tif32* ^*td*^ */prt1* ^*td*^ (*eIF3a/b Degron*)—expresses temperature sensitive degron (td) alleles of the eIF3a (TIF32) and eIF3b (PRT1) subunits (Jivotovskaya et al., 2006). Growth of this strain under restrictive conditions results in the depletion of eIF3a and eIF3b. This in turn disrupts the entire eIF3 complex, mimicking an eIF3 deletion mutant under these conditions. This disruption of the eIF3 complex further interferes with mRNA binding and 48S formation by the PIC, providing evidence for the role of eIF3 in mRNA recruitment (Jivotovskaya et al., 2006). The second mutation we investigated is a mutation to eIF3i that abrogates eIF3i binding to eIF3b: *eIF3i DDKK* (Herrmannová et al., 2012). Because the binding of both eIF3i and eIF3g to the eIF3 complex depends on this interaction, the *eIF3i DDKK* mutation mimics the absence of both subunits; purification of eIF3 from *eIF3i DDKK* cells via tagged eIF3b yields the wild-type a/b/c sub-complex (Aitken et al., 2016). The *eIF3i DDKK* mutation was previously shown to interfere with scanning and start-codon recognition *in vivo* and in cell extracts (Herrmannová et al., 2012). In addition, subsequent *in vitro* investigation demonstrated that, in the absence of eIF3i and eIF3g, the eIF3 a/b/c sub-complex is unable to promote recruitment of a natural, capped mRNA (Aitken et al., 2016).

By investigating the effects of these two mutations—which mimic the loss of either the entire eIF3 complex or two subunits of the eIF3 mRNA-entry-channel arm—we hoped to disentangle the roles of eIF3i and eIF3g from that of the entire eIF3 complex. In the presence of both mutations, we observed strong decreases in global translation levels and were able to identify mRNAs whose relative translational efficiency is either more or less sensitive—as compared to the total population of mRNAs—to each mutation. By comparing the features of these mRNAs with each other and with mRNAs sensitive to mutations targeting other initiation factors, we shed further light on the roles of eIF3 and its mRNA-entry-channel arm during mRNA recruitment. Contrasting the effects we observed when disrupting the entire eIF3 complex or targeting its mRNA-entry-channel arm disentangles the contributions of the eIF3i and eIF3g subunits from those of the other subunits of the complex. These analyses provide evidence that eIF3 and its mRNA-entry-channel arm collaborate functionally with eIF4A, eIF4B, and Ded1 to drive initiation on mRNAs with long 5′-UTRs and with a lower propensity to form stable closed-loop structures mediated by eIF4G, eIF4E, and PABP. They further reveal that eIF3 stimulates the translation of mRNAs involved in mitochondrial processes and contributes to the resolution of structurally complex regions during initial docking or scanning, and that these roles require subunits beyond eIF3i and eIF3g.

## Materials and Methods

### Cell Growth and Harvest

We created ribosome profiling libraries from eIF3 mutant and corresponding isogenic WT strains for both the *eIF3a/b Degron* (YAJ34: *MAT* ***a*** *trp1∆ leu2-3,112 ura3-52 gcn2::hisG P*_*GAL1*_ *-myc-UBR1::TRP1::ubr1 P*_*CUP1*_ *-UBI-R-HA-tif32* ^*td*^ *::URA3::tif32 P*_*CUP1*_ *-UBI-R-DHFR* ^*ts*^ *-HA-prt1* ^*td*^ *::URA3::prt1* and YAJ3: MAT**a** *trp1∆leu2-3,112 ura3-52 gcn2::hisG P*_*GAL1*_ *-myc-UBR1::TRP1::ubr1* pRS316 [*URA3*]) (Jivotovskaya et al., 2006) and *eIF3i DDKK* (H450: *MATa leu2-3,-112 ura3-52::GCN2 trp1Δ tif34Δ* hc TIF34 URA3 transformed with YCp-i/TIF34-D207K-D224K-HA or YCp-i/TIF34-HA, respectively) (Herrmannová et al., 2012) as described previously (Ingolia, 2010; Sen et al., 2015; Sen et al., 2016). We grew two biological replicates of each strain and its matching isogenic WT strain under permissive conditions before harvesting and adding to pre-warmed restrictive media for a duration resulting in an ∼90% decrease in bulk translation (as judged by polysome:monosome ratios, Supplementary Figure S1) and a final cell density at mid-log phase (OD_600_ = ∼0.6). We grew *eIF3a/b Degron* cells at 25°C in SC_Raff_ + Cu^2+^ before shifting them to pre-warmed SC_Raff/Gal_ + BCS at 36°C for 90 min. We grew *eIF3i DDKK* at 30°C in SC media before shifting them to pre-warmed SC media at 37°C for 30 min. We added cycloheximide to a final concentration of 100 μg/ml 2 min prior to harvesting by filtration through a Kontes filtration apparatus and flash freezing in liquid nitrogen with 2 ml of ribosome footprinting buffer (20 mM Tris pH 8.0, 140 mM KCl, 1.5 mM MgCl2, 1% Triton, 100 μg/ml cycloheximide).

### Ribosome Profiling and RNA-Seq Library Preparation

We generated sequencing libraries of ribosome footprints and total mRNA as previously described (Ingolia, 2010; Sen et al., 2015; Sen et al., 2016). Briefly, we lysed cells using a freezer mill and then prepared lysates by centrifuging 5 min at 3,000 × g, collecting the supernatant and then centrifuging 12 min at >20,000 × g. We then collected the supernatant and flash-froze in 30 OD_260_ aliquots. We purified ribosome footprints by adding 5 µL RNase1 to one aliquot of purified lysate and incubating 60 min at 26°C with mixing at 700 rpm. We then added 5 µL SuperAsin (Thermo Fisher) and loaded on a 10–50% sucrose gradient and centrifuged at 40,000 rpm for 3 h and then collected the monosome peak using a gradient fractionator. We then purified RNA from purified monosomes via hot phenol extraction. We purified total mRNA from one 30 OD_260_ aliquot of purified lysate using the miRNeasy mini kit (Qiagen) per the vendor’s instructions and then randomly fragmented at 70°C for 8 min using Fragmentation Reagent (Invitrogen). We then performed subsequent steps (size selection, linker ligation, reverse transcription, circularization, rRNA subtraction, and PCR amplification) as previously described and had libraries sequenced using an Illumina HiSeq system.

### Analysis of Sequencing Data

We processed and analyzed sequencing libraries of ribosome footprints and total mRNA as described previously (Ingolia, 2010; Sen et al., 2015; Sen et al., 2016). We then employed DESeq2 (Love et al., 2014) for statistical analysis of differences in ribosome footprint and RNA-seq read counts, and TE_rel_ values between WT and mutant samples, as previously reported (Martin-Marcos et al., 2017; Kulkarni et al., 2019). We excluded genes with fewer than 128 total mRNA reads in the four samples combined (two replicates of both WT and mutant strains) from the calculation of TE_rel_ values. We then performed subsequent analysis of mRNA features and characteristics within R, using custom scripts, together with previously-reported datasets reporting 5′-UTR lengths, PARS values, closed-loop-forming propensity of individual mRNAs, previously identified uORFs, or ∆TE_rel_ values observed in the presence of mutations targeting eIF4A, eIF4B, Ded1, eIF1, or eIF1A. We performed Gene Ontology analysis using the Gene Ontology Resource PANTHER classification system. Statistical tests were performed as described in the main text and figures.

## Results

### Disruption of the eIF3 Complex Provokes Severe Translational Defects

**FIGURE 1:**
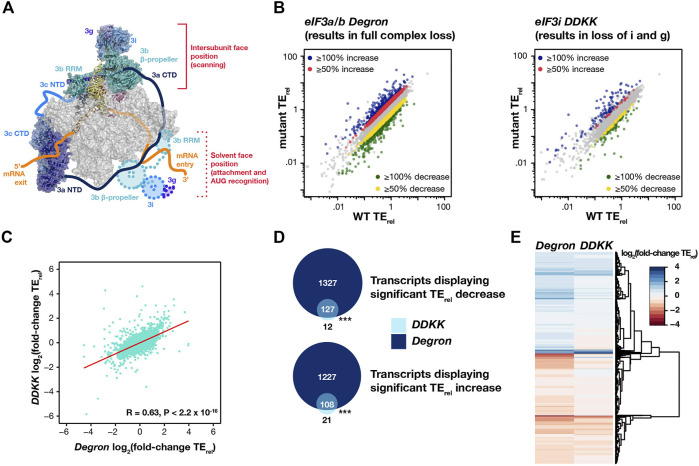
Disruption of the eIF3 complex or its mRNA-entry channel arm provoke strong and overlapping effects on the translation of mRNAs across the transcriptome. **(A)** Structural model of eIF3 bound to the PIC (PDB 6GSM) viewed looking down at top of the small (40S) ribosomal subunit head so as to visualize the path of mRNA as it enters and exits the PIC (Llácer et al., 2021). The small (40S) ribosomal subunit is shown in grey, and the initiator tRNA and mRNA are shown in yellow and orange, respectively (with the path of the mRNA entering and exiting the PIC shown as a cartoon). Subunits of the eIF3 complex are shown in blues and greens, with the mRNA-entry-channel arm shown in two positions: at the intersubunit face of the PIC (identified density seen in this structure) or at the solvent face (cartoons depicting approximate location observed in structures lacking mRNA or after start-codon recognition). **(B)** TE_rel_ correlation plots comparing observed TE_rel_ values in either *eIF3a/b Degron* (left) or *eIF3i DDKK* (right) cells with TE_rel_ values observed in isogenic wild-type cells. Transcripts displaying significant (P_adj_ < 0.05) TE_rel_ increases (≥50% red and ≥100% blue) or decreases (≥50% yellow and ≥100% green) as determined by DESeq2 analysis are shown in color. **(C)** Correlation plot comparing observed TE_rel_ changes in *eIF3a/b Degron* and *eIF3i DDKK* cells, with predicted Pearson correlation shown in red. **(D)** Venn diagrams describing the overlap in transcripts displaying significant TE_rel_ decreases (top) or increases (bottom) in *eIF3a/b Degron* (dark blue) or *eIF3i DDKK* (light blue) cells. *** = P < 10^−10^, ** = P < 10^−5^, * = P < 0.05, HyperGeometric test. **(E)** Heatmap and dendrogram resulting from hierarchical clustering analysis of significant TE_rel_ changes observed in *eIF3a/b Degron* and *eIF3i DDKK* cells.

To investigate the transcriptome-wide roles of eIF3, we performed ribosome profiling in two *S. cerevisiae* strains in which the eIF3 complex is partially or entirely compromised. The *eIF3a/b Degron* strain expresses temperature-sensitive degron variants of the eIF3a and eIF3b subunits. Depletion of these subunits provokes the loss of the entire eIF3 complex (Jivotovskaya et al., 2006). The *eIF3i DDKK* strain expresses a variant of the eIF3i subunit that is unable to bind stably to eIF3b (Herrmannová et al., 2012). Because both eIF3i and eIF3g depend on this interaction to associate with the remainder of the eIF3 complex, this results in the loss of both subunits, which normally contribute to the mRNA-entry-channel arm of eIF3 (Figure 1A). This arm has been observed in two distinct locations of the PIC, depending on its functional state (Llácer et al., 2015; Llácer et al., 2018).

Before constructing ribosome profiling libraries, we first investigated the effects of the *eIF3a/b Degron* and *eIF3i DDKK* mutations on global translation levels, as assayed by polysome profiling. For each strain, we grew cells first under permissive conditions and then shifted them to restrictive conditions for 30, 60, 90, and 120 min. Both strains exhibit no growth defect under permissive conditions but manifest severe growth defects at restrictive conditions (Jivotovskaya et al., 2006; Herrmannová et al., 2012). Consistent with this, polysome profiles collected under permissive growth conditions for both strains were similar to those collected for isogenic wild-type strains (Supplementary Figure S1A). In contrast, we observed strong decreases in polysome to monosome ratios (P/M) upon shifting to restrictive conditions for both strains, with *eIF3a/b Degron* and *eIF3i DDKK* cells exhibiting an approximately 90% decrease in P/M (as compared to isogenic WT cells grown under the same conditions) after 30 min and 90 min at restrictive conditions, respectively (Supplementary Figure S1B,C).

### Relative TE Changes Identify mRNAs Most or Least Sensitive to the Disruption of the Entire eIF3 Complex or Its mRNA-Entry-Channel Arm

Given the marked decrease in global translation levels we observed in both strains, we next asked how these global affects translate to individual mRNAs across the transcriptome. To that end, we constructed ribosome profiling and RNA-seq libraries from both the *eIF3i DDKK* and *eIF3a/b Degron* strains (and their corresponding isogenic WT strains) grown under restrictive conditions and calculated relative translational efficiency (TE_rel_) values for coding sequences (CDS), ignoring reads obtained from the initial 15 codons and from the final 5 codons to avoid cycloheximide-induced artifacts (Gerashchenko and Gladyshev, 2014). Owing to the absence of an internal read-count standard, read counts from both ribosome profiling and RNA-seq libraries are normalized to the total library size for each condition. The TE_rel_ values we calculate from these normalized read counts thus do not enable direct comparison of absolute TE between samples. Instead, TE_rel_ values provide a measure of the translational status of individual mRNAs as compared to the overall population of mRNAs within each sample. We also calculated TE_rel_ values for a set of previously identified uORFs (Martin-Marcos et al., 2017; Kulkarni et al., 2019) but did not attempt to identify novel translated uORFS owing to the inclusion of cycloheximide in our library preparation. Both ribosome footprint and RNA-seq libraries were highly reproducible across replicates for each condition (Supplementary Figure S2).

Using the R DESeq2 package (Love et al., 2014), we then identified transcripts whose TE_rel_ was significantly changed (P_adj_ < 0.05) in each strain, as compared to an isogenic WT strain (Figure 1B). Owing to the marked decrease in global translational levels we observed in each mutant strain, as well as the normalization to total ribosomal footprint reads performed when calculating TE_rel_, we interpreted mRNAs exhibiting significant TE_rel_ decreases as having a greater than average dependence on the either the eIF3i and eIF3g subunits lost in *eIF3i DDKK* cells or on the entire eIF3 complex disrupted in *eIF3a/b Degron* cells. We interpreted those mRNAs exhibiting significant TE_rel_ increases as instead having a weaker than average dependance on the regions of the eIF3 complex targeted by each mutation. The significant effects on global translational levels that we observed in both *eIF3i DDKK* and *eIF3a/b Degron* cells in fact suggest that most mRNAs likely experience decreases in their absolute TE. Nonetheless, comparison of these significant changes in TE_rel_ (∆TE_rel_) enables identification of those mRNAs whose translation is most or least sensitive to disruption of eIF3 or its mRNA-entry-channel arm in a background where global translational levels are repressed.

In *eIF3a/b Degron* cells—in which the entire eIF3 complex is disrupted—we identified 1,455 transcripts whose TE_rel_ decreased and 1,340 transcripts whose TE_rel_ increased (5,466 total with significant read counts), as compared to TE_rel_ values in an isogenic WT strain (Figure 1B). Because eIF3 has been implicated in mediating the translation of specific mRNAs in a number of cell types (Sha et al., 2009; Lee et al., 2015; Rode et al., 2018; Lin et al., 2020; Lamper et al., 2020), we investigated the gene ontology (GO) terms associated with these affected mRNAs. The set of transcripts whose TE_rel_ decreased in *eIF3a/b Degron* cells was enriched for mRNAs with GO terms involved in mitochondrial translation and gene expression, as well as a variety of metabolic processes (Supplementary Figure S3). These most sensitive mRNAs were under-enriched in mRNAs involved in RNA processing and ribosome biogenesis. Consistent with this, mRNAs whose TE_rel_ increased in *eIF3a/b Degron* cells were enriched in GO terms associated with RNA processing and under-enriched in terms associated with mitochondrial translation and gene expression (Supplementary Figure S3).

In *eIF3i DDKK* cells—in which the eIF3i and eIF3g subunits of the eIF3 mRNA-entry-channel arm are lost from the complex—we identified 139 transcripts whose TE_rel_ decreased and 133 transcripts whose TE_rel_ increased, as compared to TE_rel_ values in an isogenic WT strain (Figure 1B). We did not observe any significant over- or under-enrichment of specific GO terms in affected transcripts in these cells, perhaps because the strong global effects on translation in *eIF3i DDKK* cells are more uniformly distributed amongst all mRNAs, resulting in widespread but uniform decreases in absolute TE levels across the transcriptome with more limited effects on the relative TE of individual mRNAs.

In both *eIF3i DDK*K and *eIF3a/b Degron* cells, we observed strong increases in uORF translation (Supplementary Figure S4A). Of 4,830 uORFs identified previously, 529 displayed significant (P_adj_ < 0.05) increases in TE_rel_ in *eIF3a/b Degron* cells, whereas 21 displayed significant decreases in TE_rel_. In *eIF3i DDKK* cells, 391 uORFs displayed significant (P_adj_ < 0.05) increases in TE_rel_, whereas 5 displayed significant decreases in TE_rel_. This global increase in uORF translation in both strains is likely a result of the previously described effects of cycloheximide on read counts near the start codon (Gerashchenko and Gladyshev, 2014), which cannot be discarded as in the calculation of ORF TE_rel_ values owing to the short length of these regions. Thus we focused subsequent analysis of uORF TE_rel_ values on any observed differential behavior between uORFs within the same strain.

Because the eIF3 complex is either partly or entirely disrupted in both cell lines, we investigated the degree to which the TE_rel_ effects we observed were similar in *eIF3i DDKK* and *eIF3a/b Degron* cells. We observed a significant correlation (R = 0.63, P < 2.2 × 10^−16^) between the ∆TE_rel_ values observed in both strains (Figure 1C). Additionally, we found a strong overlap between those transcripts exhibiting TE_rel_ decreases in *eIF3i DDKK* and *eIF3a/b Degron* cells (Figure 1D), with the changes observed in *eIF3i DDKK* cells appearing to represent a subset of the changes observed in *eIF3a/b Degron* cells. Moreover, the overall portfolio of ∆TE_rel_ values we observe is similar for both strains, as evidenced by heat map comparison of the magnitude and direction of observed changes (Figure 1E). Together, these results are consistent with strong translational effects upon disruption of the eIF3 mRNA-entry-channel arm (as in *eIF3i DDKK* cells) or depletion of the entire eIF3 complex (as in *eIF3a/b Degron* cells) and a role for eIF3 in promoting the translation of mRNAs with mitochondrial roles.

### mRNAs Most Sensitive to the *eIF3a/b Degron* or *eIF3i DDKK* Mutations Possess Longer 5′-Untranslated Regions

**FIGURE 2:**
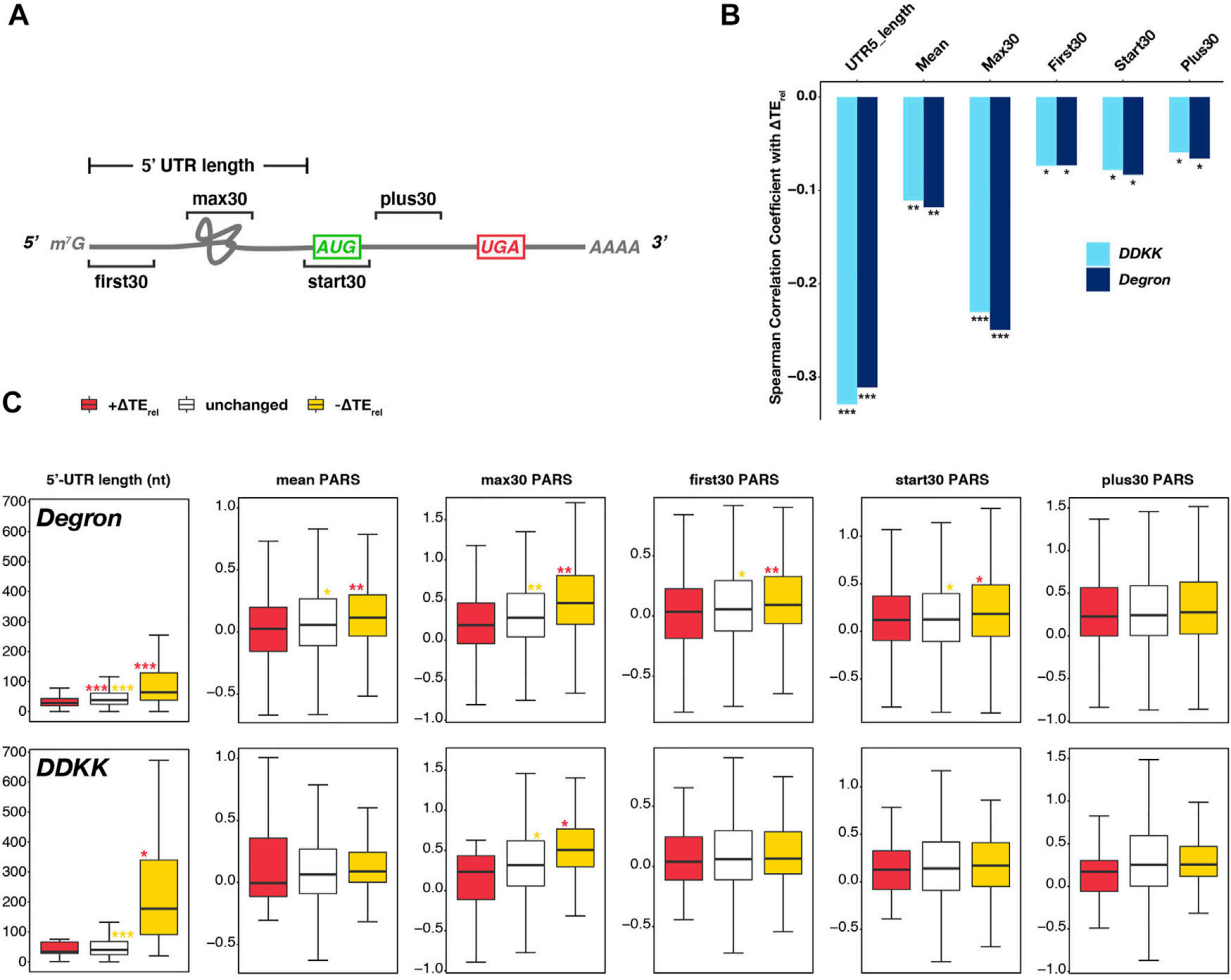
Transcripts most sensitive to disruption of the eIF3 complex or its mRNA-entry-channel arm possess longer 5′-UTRs. **(A)** Cartoon depicting a hypothetical mRNA and detailing the 5′-UTR length and the specific 30-nt windows within which mean PARS values (Kertesz et al., 2010) were calculated as measures of structural complexity. **(B)** Bar plot comparing the Spearman correlation coefficients obtained when comparing observed ∆TE_rel_ values in each mutant eIF3 strain and different measures of 5′-UTR length or complexity. *** = P < 10^−10^, ** = P < 10^−5^, * = P < 0.05. **(C)** Box and whisker plots comparing different measures of 5′-UTR length or complexity between mRNAs whose TE_rel_ significantly increases (red), decreases (yellow), or is not significantly changed (white) in *eIF3a/b Degron* (top row) or *eIF3i DDKK* (bottom row) cells. *** = P_adj_ < 10^−10^, ** = P_adj_ < 10^−5^, * = P_adj_ < 0.05, color indicates comparison set; Wilcoxon Test for 5′-UTR lengths, ANOVA with post-hoc Tukey test for others.

We next investigated the structural features (Figure 2A) of mRNAs most or least sensitive to the *eIF3i DDKK* and *eIF3a/b Degron* mutations, as identified by DESeq2 analysis of TE_rel_ changes in each strain. eIF3 is required for the overall process of mRNA recruitment both *in vivo* (Jivotovskaya et al., 2006) and *in vitro* (Mitchell et al., 2010; Aitken et al., 2016), and mutations to several eIF3 subunits elicit defects in mRNA recruitment or its component events of initial docking, scanning, and start-codon recognition (Valášek et al., 2002; Nielsen et al., 2004; Chiu et al., 2010; Cuchalova et al., 2010; Elantak et al., 2010; Herrmannová et al., 2012).

To shed light on the contribution of the eIF3 complex and the eIF3i and eIF3g subunits of the mRNA-entry-channel arm to scanning processivity, we first asked if the ∆TE_rel_ values we observed in each strain correlated with 5′-UTR length. Upon restricting our analysis to mRNAs previously shown to have a dominant 5′-UTR isoform (defined as mRNAs for which one isoform accounts for at least 40% of all transcripts and is present at an abundance at least twice that of the next most abundant isoform) (Pelechano et al., 2013; Zinshteyn et al., 2017), we observed significant (P < 10^−59^) negative correlations between 5′-UTR length and ∆TE_rel_ in both the *eIF3i DDKK* and *eIF3a/b Degron* strains (Figure 2B). Consistent with this effect, we also observed a negative correlation between ∆TE_rel_ and distance from the 5′ end for a set uORFs identified in previous studies (Martin-Marcos et al., 2017; Kulkarni et al., 2019), though this correlation was significant only in *eIF3i DDKK* cells (Supplementary Figure S4B).

To interrogate the roles of eIF3 and the eIF3i and eIF3g subunits in resolving structural impediments during initial mRNA docking or scanning, we next determined the relationship between ∆TE_rel_ and the propensity of specific regions of an mRNA to form secondary structures, as measured by their differential sensitivity *in vitro* to nucleases specific for single- or double-stranded RNA (PARS, Figure 2A) (Kertesz et al., 2010). In *eIF3a/b Degron* and *eIF3i DDKK* cells, we observed a significant (P < 10^−8^ and P < 10^−7^, respectively) negative correlation between ∆TE_rel_ and mean 5′-UTR PARS values, as well as with mean PARS scores determined within specific 30 nucleotide (nt) windows located at the first 30 nt of the 5′-UTR (First30; P < 10^−3^ for both), 30 nt centered around the AUG start codon (Start30; P < 10^−4^ for both), and the first 30 nt downstream of the Start30 window (Plus30; P < 10^−3^ and P < 10^−2^, respectively) (Figure 2B) (Sen et al., 2015; Sen et al., 2016). Of the correlations we observed between ∆TE_rel_ and PARS measures, the strongest and most significant was with the maximum PARS score observed within any 30 nt window within the 5′-UTR (Max30; P < 10^−32^ and P < 10^−38^, respectively) (Figure 2B).

Having observed these correlations between ∆TE_rel_ and 5′-UTR length or structural complexity in both the *eIF3i DDKK* and *eIF3a/b Degron* strains, we next investigated if we could detect significant differences in the median values of these measures when comparing mRNAs whose TE_rel_ was either significantly decreased or increased in either strain (Figure 2C). As before, we restricted our analysis to mRNAs with one dominant 5′ isoform. In *eIF3a/b Degron* cells, mRNAs displaying significant negative ∆TE_rel_ values possess longer 5′-UTRs and those displaying significant positive ∆TE_rel_ values possess shorter 5′-UTRs, as compared to mRNAs whose TE_rel_ was not significantly affected (P_adj_ < 10^−28^ for all pairwise comparisons, Wilcoxon test). Similarly, in *eIF3i DDKK* cells, mRNAs displaying significant negative ∆TE_rel_ values possess longer 5′-UTRs than both unaffected mRNAs (P_adj_ < 10^−18^) and mRNAs displaying significant positive ∆TE_rel_ values (P_adj_ < 10^−3^). However, we did not observe a significant difference in 5′-UTR lengths when comparing mRNAs displaying significant positive ∆TE_rel_ values and unaffected mRNAs in *eIF3i DDKK* cells, perhaps because the set of mRNAs expressed as a dominant transcript isoform and exhibiting significant positive ∆TE_rel_ values in these cells is relatively small (n = 57). In both cell lines, we observed similar results when comparing 5′-UTR values reported in a separate study (Kertesz et al., 2010) (Supplementary Figure S5A).

When comparing measures of structural complexity (as measured by PARS values across the 5′-UTR and in distinct windows), we again observed differences between mRNAs whose TE_rel_ either increased or decreased in *eIF3a/b Degron* cells. mRNAs displaying significant TE_rel_ decreases in these cells have higher mean and max30 5′-UTR PARS values, as compared to unaffected mRNAs (P_adj_ < 10^−3^ and P_adj_ < 10^−6^, respectively; ANOVA and post-hoc Tukey test) and mRNAs displaying significant TE_rel_ increases (P_adj_ < 10^−9^ and P_adj_ < 10^−6^, respectively). Similarly, mRNAs whose TE_rel_ decreased in *eIF3a/b Degron* cells have higher PARS values at the 5′ end of their 5′-UTRs (first30) and around their start codons (start30) as compared to mRNAs whose TE_rel_ either increased (P_adj_ < 10^−7^ and P_adj_ < 10^−2^, respectively) or was unaffected (P_adj_ < 10^−2^ for both) in these cells. However, these differences appear more modest than those observed for 5′-UTR length, mean and max30 PARS values. In contrast, there is no significant difference in the PARS values downstream of the start-codon window (plus30) between mRNAs whose TE_rel_ either increased, decreased, or was unaffected in *eIF3a/b Degron* cells.

In *eIF3i DDKK* cells, we did not observe significant differences in most measures of 5′-UTR structural complexity when comparing mRNAs whose TE_rel_ either decreased, increased, or was unaffected. The one exception is the max30 PARS values of mRNAs whose TE_rel_ decreased in these cells, which is higher than for mRNAs whose TE_rel_ was not significantly affected (P_adj_ < 10^−3^) and modestly different than for transcripts whose TE_rel_ increased in these cells (P_adj_ < 0.02), perhaps because the number of transcripts displaying significant TE_rel_ increases in these cells and with available PARS scores is limited (n = 17). Nonetheless, these observations are consistent with a role for eIF3 and its mRNA-entry-channel arm in processive scanning through longer 5′-UTRs. eIF3 also appears to contribute to resolving structural complexity within the 5′-UTR during initial docking or scanning, though the mRNA-entry-channel arm may play a more peripheral role in these events.

### Disruption of the eIF3 mRNA-Entry-Channel Arm Exerts Modest Effects on Discrimination Against Start Codons in Poor Kozak Sequence Context

Because various subunits of eIF3 appear to play roles in start-codon recognition (Nielsen et al., 2004; Valasek et al., 2004; Chiu et al., 2010; Cuchalova et al., 2010; Herrmannová et al., 2012), we next asked whether there was a correlation between the observed ∆TE_rel_ values in the *eIF3i DDKK* and *eIF3a/b Degron* strains and the strength of the Kozak consensus sequence surrounding the AUG start codon for each mRNA. However, we did not observe a significant correlation between context scores (calculated for nucleotides −6 to +4) and ∆TE_rel_ values in either *eIF3i DDKK* or *eIF3a/b Degron* cells (Supplementary Figure S6).

**FIGURE 3:**
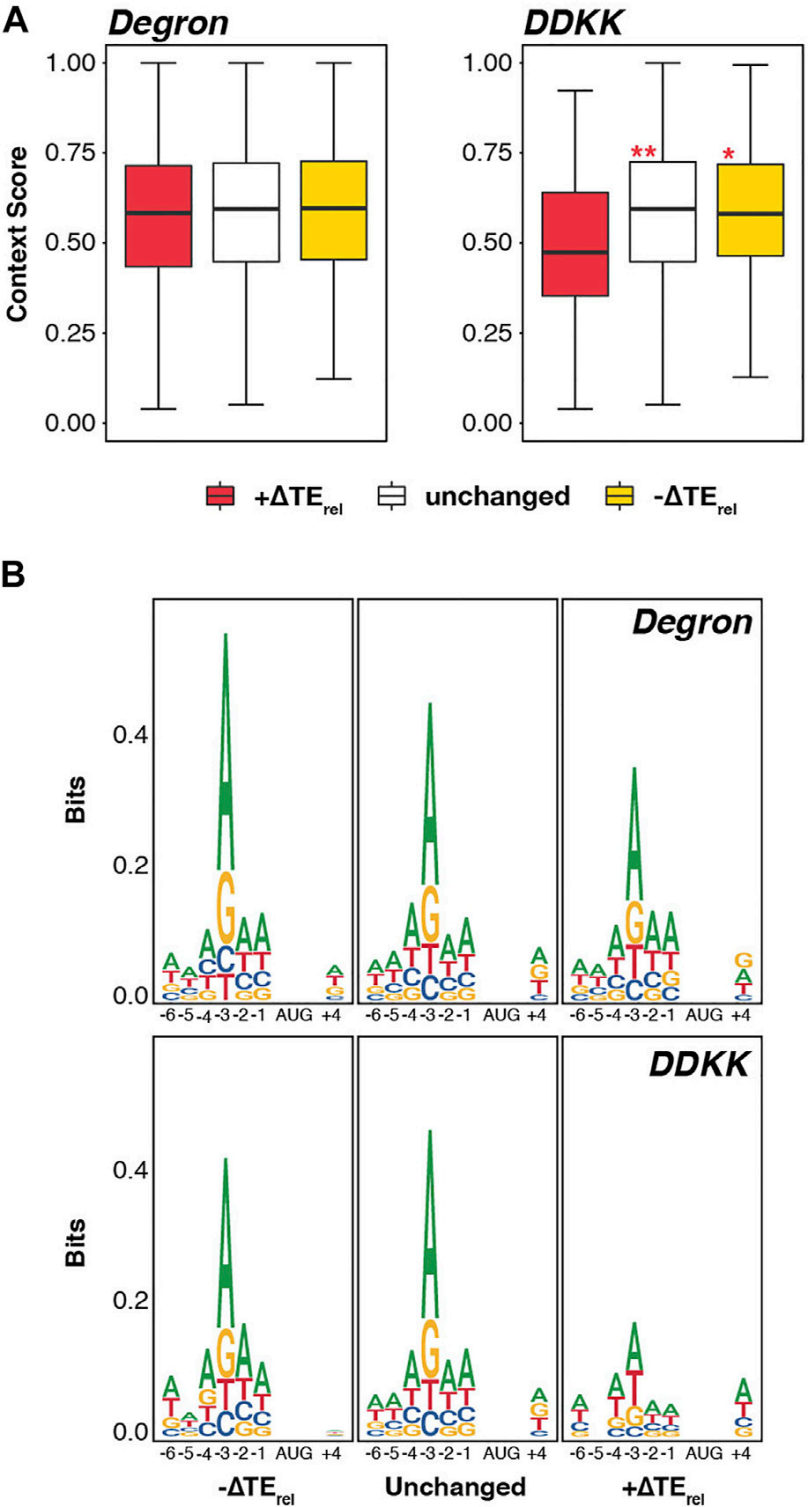
Disruption of the eIF3 mRNA-entry-channel arm interferes with discrimination against start codons appearing in poor Kozak sequence context. **(A)** Box and whisker plots comparing the Kozak sequence context (nt -6 thru +4) of transcripts whose TE_rel_ significantly increases (red), decreases (yellow), or is not significantly changed (white) in each eIF3 mutant strain. *** = P_adj_ < 10^−10^, ** = P_adj_ < 10^−5^, * = P_adj_ < 0.05, color indicates comparison set; ANOVA with post-hoc Tukey test. **(B)** Sequence logos of nt -6 to +4 of transcripts whose TE_rel_ significantly increases, decreases, or is not significantly changed in each eIF3 mutant strain.

Consistent with this, we observed no significant difference in the median context scores for mRNAs whose TE_rel_ either increased, decreased, or was unaffected in *eIF3a/b Degron* cells (Figure 3A). Nonetheless, we did observe modest but significant differences between the median context scores of mRNAs displaying TE_rel_ increases in *eIF3i DDKK* cells, which are weaker than either those of mRNAs displaying TE_rel_ decreases or those whose TE_rel_ was unaffected (P_adj_ < 10^−4^ and P_adj_ < 10^−5^, respectively).

Given these modest effects, we investigated the sequence logos in the vicinity of the start codons of these distinct mRNAs (Figure 3B). Here again, we observed no difference when comparing mRNAs whose TE_rel_ either increased, decreased, or was unaffected in *eIF3a/b Degron* cells. However, consistent with the differences we observed in context scores in *eIF3i DDKK* cells, mRNAs displaying TE_rel_ increases in this background show a weaker preference for an adenine at the −3 nt than do mRNAs whose TE_rel_ is either unaffected or decreases; optimal Kozak consensus sequences contain a purine base (most often adenine) at position −3 and a guanosine at position +4 (Hinnebusch, 2014). Together, these results suggest a peripheral role for the eIF3i and eIF3g subunits of the mRNA-entry-channel arm in discriminating against start codons in poor Kozak context.

### The Transcriptome-Wide Effects of the *eIF3a/b Degron* and *eIF3i DDKK* Mutations Most Closely Resemble Those Observed for Mutations Targeting Factors Involved in mRNA Recruitment

In light of previous work implicating eIF3 in both mRNA recruitment and start-codon recognition and our results here, we next compared the effects we observed in *eIF3a/b Degron* and *eIF3i DDKK* cells to those previously reported for mutations targeting other initiation factors. The effects of mutations targeting eIFA, eIF4B, and Ded1—which appear to contribute either to initial PIC docking or scanning—have previously been investigated using ribosome profiling (Sen et al., 2015, 2016). Specifically, these studies investigated the effect of temperature-sensitive alleles of eIF4A and Ded1, and a deletion of eIF4B. Importantly, the sequencing libraries from which these datasets were obtained were prepared by addition of cycloheximide to media in which cells exhibited strong translational defects, as were our sequencing libraries.

**FIGURE 4:**
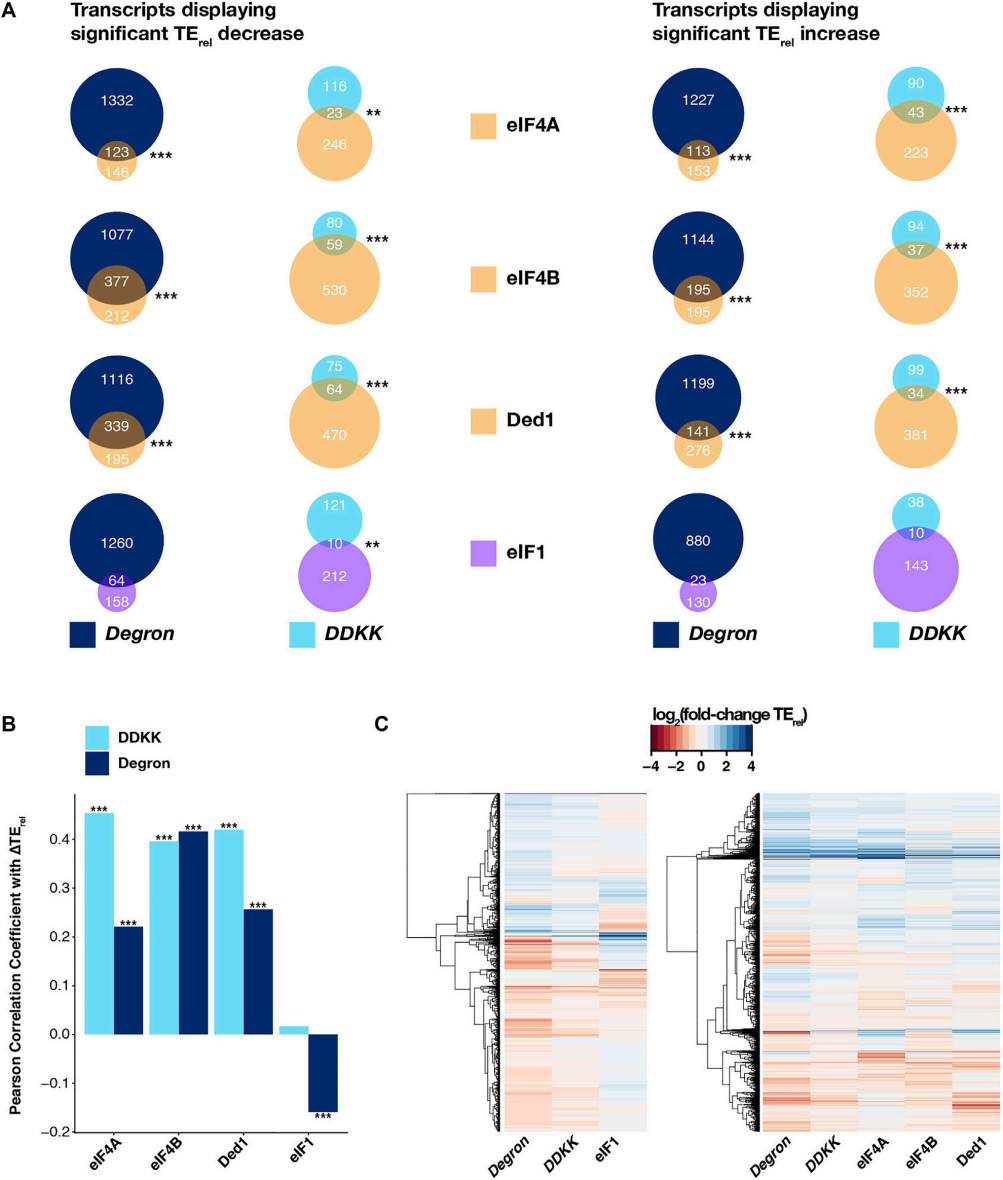
The effects of disruption of the entire eIF3 complex or its mRNA-entry-channel arm are similar to those observed for mutations targeting other initiation factors involved in mRNA recruitment. **(A)** Venn diagrams showing the overlap between mRNAs displaying either TE_rel_ decreases (left) or increases (right) in *eIF3a/b Degron* (dark blue) or *eIF3i DDKK* (light blue) cells and cells expressing mutations targeting other initiation factors that contribute to mRNA recruitment (eIF4A, eIF4B, and Ded1; light orange) or to start-codon recognition (eIF1, purple). *** = P < 10^−10^, ** = P < 10^−5^, * = P < 0.05, HyperGeometric test. **(B)** Bar plot showing Pearson correlation coefficients obtained when comparing ∆TE_rel_ values observed in *eIF3a/b Degron* (dark blue) or *eIF3i DDKK* (light blue) cells and those observed in cells expressing mutations targeting eIF4A, eIF4B, Ded1, or eIF1. *** = P < 10^−10^, ** = P < 10^−5^, * = P < 0.05. **(C)** Heatmap and dendrograms resulting from hierarchical clustering analysis of significant TE_rel_ changes observed in *eIF3a/b Degron* and *eIF3i DDKK* cells and cells with mutations targeting either eIF1 (left) or eIF4A, eIF4B, and Ded1 (right).

We found significant overlaps (Figure 4A, left panel) in the specific mRNAs experiencing significant TE_rel_ decreases in *eIF3i DDKK* or *eIF3a/b Degron* cells and those mRNAs whose TE_rel_ was significantly decreased in the presence of mutations targeting eIF4A (P < 10^−6^ and P < 10^−19^ for *eIF3i DDKK* and *eIF3a/b Degron*, respectively; HyperGeometric test), eIF4B (P < 10^−20^ for both), and Ded1 (P < 10^−20^ and P < 10^−11^). We similarly found significant overlaps when comparing the sets of mRNAs whose TE_rel_ increased in the presence of these mutations (eIF4A, P < 10^−20^ and P < 10^−17^; eIF4B, P < 10^−13^ and P < 10^−20^; Ded1, P < 10^−10^ and P < 10^−11^). Consistent with these overlapping effects, we also observed significant positive correlations between the ∆TE_rel_ values we observed in both *eIF3i DDKK* and *eIF3a/b Degron* cells and ∆TE_rel_ values observed in the presence of mutations targeting eIF4A, eIF4B, and Ded1 (Figure 4B and Supplementary Figure S7). Global comparison of the magnitude and direction of observed ∆TE_rel_ values from these distinct experiments further revealed a similar transcriptome-level portfolio of effects (Figure 4C, right panel).

Having observed these similarities, we next compared the effects observed in *eIF3i DDKK* and *eIF3a/b Degron* cells to those obtained from cells expressing an eIF1 variant (*L96P*) that increases recognition of both AUG start codons in poor context and near-cognate uORF start codons (Zhou et al., 2020). The sequencing libraries giving rise to this dataset were also prepared under conditions similar to those we employed in our experiments. In contrast to the significant overlaps we observed when comparing to datasets from eIF4A, eIF4B, or Ded1 mutant cells, we only found a significant overlap between mRNAs displaying TE_rel_ decreases in *eIF3i DDKK* and *L96P eIF1* cells (P < 10^−5^) and not in *eIF3a/b Degron* cells or for mRNAs displaying TE_rel_ increases in either eIF3 background (Figure 4A). Consistent with this, we observed a significant but negative correlation between ∆TE_rel_ values from *eIF3a/b Degron* and *L96P eIF1* cells and no significant correlation between *eIF3i DDKK* and *L96P eIF1* cells (Figure 4B and Supplementary Figure S7). Moreover, global comparison of the observed ∆TE_rel_ values from these datasets further revealed a distinct pattern of transcriptome-wide effects in *L96P eIF1* cells when compared to either *eIF3i DDKK* or *eIF3a/b Degron* cells (Figure 4C, left panel). We also observed weak but modestly significant correlations in ∆TE_rel_ values when comparing our eIF3 datasets to a ribosome profiling dataset obtained from cells expressing *R13P eIF1A*, in which discrimination against near-cognate codons or AUG codons in poor context was increased (Supplementary Figure S7). However, the sequencing libraries for this dataset were prepared from cells harvested in the absence of cycloheximide, which complicates their comparison to our sequencing results.

Together with the more pronounced overlap we observed when comparing our datasets with those obtained from cells expressing mutant versions of eIF4A, eIF4B, and Ded1, these results suggest that eIF3 and its mRNA-entry-channel arm contribute to initial docking and scanning of the mRNA, whereas their contributions to start-codon recognition may be less critical or peripheral to those of eIF1 and eIF1A.

### Long Transcripts Less Likely to Form Closed Loop Structures Are More Sensitive to Disruption of the Entire eIF3 Complex or Its mRNA-Entry-Channel Arm

**FIGURE 5:**
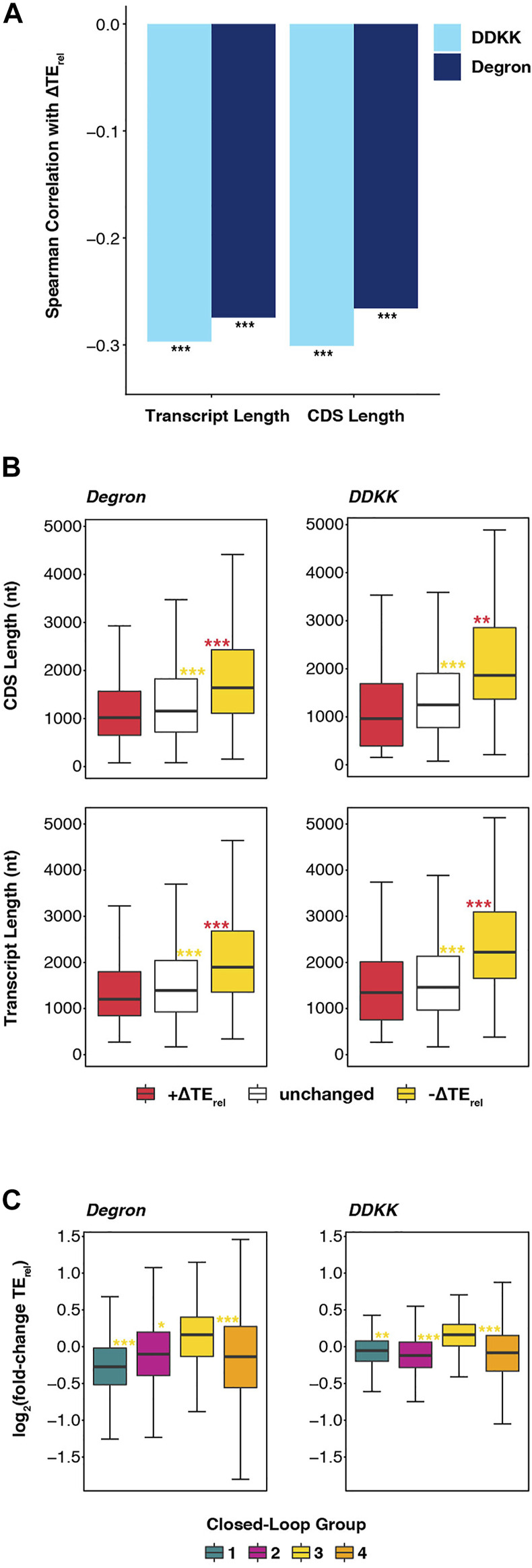
Disruption of the eIF3 complex or its mRNA-entry-channel arm most strongly affect long mRNAs with a weaker dependence on closed loop formation. **(A)** Bar plot showing Spearman correlation coefficients obtained when comparing ∆TE_rel_ values observed in *eIF3a/b Degron* (dark blue) or *eIF3i DDKK* (light blue) cells with overall transcript length and CDS length *** = P < 10^−10^, ** = P < 10^−5^, * = P < 0.05. **(B)** Box and whisker plots comparing the CDS and overall transcript length of mRNAs whose TE_rel_ significantly increases (red), decreases (yellow), or is unaffected (white) in either *eIF3a/b Degron* or *eIF3i DDKK* cells. *** = P_adj_ < 10^−10^, ** = P_adj_ < 10^−5^, * = P_adj_ < 0.05, color indicates comparison set; Wilcoxon test. **(C)** Box and whisker plots comparing the TE_rel_ changes observed in either *eIF3a/b Degron* (left) or *eIF3i DDKK* (right) cells for previously identified groups of transcripts (Costello et al., 2015) that differentially associate with closed-loop factors such as eIF4G, eIF4E, and PABP. *** = P_adj_ < 10^−10^, ** = P_adj_ < 10^−5^, * = P_adj_ < 0.05, color indicates comparison set; ANOVA and post-hoc Tukey test.

Because of the similarities we observed in the effects of *eIF3i DDKK* and *eIF3a/b Degron* mutations and mutations targeting eIF4A, eIF4B, and Ded1, we investigated whether these similarities extended to the observation that long transcripts and transcripts with lower closed-loop-forming potential are particularly sensitive to deletion of eIF4B or to mutations of eIF4A or Ded1 (Sen et al., 2016). In fact, we observed significant negative correlations between ∆TE_rel_ and both overall transcript length and coding sequence (CDS) length in both *eIF3i DDKK* and *eIF3a/b Degron* cells (Figure 5A). Comparing the overall length of mRNAs whose TE_rel_ decreased in each mutant eIF3 background reveals them to be significantly longer than both unaffected mRNAs (P_adj_ < 10^−13^ and P_adj_ < 10^−16^ for *eIF3i DDKK* and *eIF3a/b Degron*, respectively; Wilcoxon test) and mRNAs whose TE_rel_ increased (P_adj_ < 10^−14^ and P_adj_ < 10^−16^) (Figure 5B). Similarly, the CDS lengths of mRNAs whose TE_rel_ decreased in the presence of each eIF3 mutation is longer than both unaffected mRNAs (P_adj_ < 10^−11^ and P_adj_ < 10^−16^) and mRNAs whose TE_rel_ increased (P_adj_ < 10^−5^ and P_adj_ < 10^−16^).

We further observed that, as in cells expressing mutations of eIF4A or Ded1 or in which the gene coding for eIF4B was deleted, mRNAs identified in a previous study (Costello et al., 2015) as having strong closed-loop-forming potential owing to their enrichment in eIF4G, eIF4E, and PABP (group 3) are less sensitive than other mRNAs to both the *eIF3i DDKK* (P_adj_ < 10^−7^; P_adj_ < 10^−14^; and P_adj_ < 10^−14^ for comparison to groups 1, 2, and 4 respectively) or *eIF3a/b Degron* (P_adj_ < 10^−12^; P_adj_ < 10^−4^; and P_adj_ < 10^−15^) mutations (Figure 5C). Moreover, we observed positive median ∆TE_rel_ values for strong closed-loop mRNAs (group 3) and negative median ∆TE_rel_ values for weak closed-loop mRNAs (groups 1 and 2), suggesting that mRNAs with strong closed-loop-forming potential compete more effectively for the initiation machinery when eIF3 or its mRNA-entry-channel arm are disrupted, whereas mRNAs less likely to form closed-loop structures are disadvantaged under these conditions. Taken together, these results suggest that, like eIF4A, eIF4B, and Ded1, eIF3 and its mRNA-entry-channel arm may contribute to driving initiation on long mRNAs less likely to form eIF4G-, eIF4E-, and PABP-dependent closed loop structures *in vivo*. While the translation of most mRNAs likely depends on contributions from these factors, translation of these long and closed-loop-dependent mRNAs is particularly sensitive to their disruption.

### Comparing the mRNAs Sensitive to Both eIF3 Mutations With Those Uniquely Sensitive to the *eIF3a/b Degron* Mutation Provides Clues to the Roles of the eIF3i and eIF3g Subunits

Because the *eIF3i DDKK* and *eIF3a/b Degron* mutations mimic the loss of either the eIF3i and eIF3g subunits (*eIF3i DDKK*) or the entire eIF3 complex (*eIF3a/b Degron*), we reasoned that comparing those mRNAs whose TE_rel_ was affected uniquely in *eIF3a/b Degron* cells to those whose TE_rel_ was affected in both *eIF3a/b Degron* and *eIF3i DDKK* cells might disentangle the roles of these distinct regions of the eIF3 complex. mRNAs whose TE_rel_ was affected in both cell lines might depend more heavily on the contributions of the eIF3i and eIF3g subunits of the eIF3 mRNA-entry-channel arm, whereas mRNAs whose TE_rel_ was affected solely in *eIF3a/b Degron* cells might depend more heavily on the contributions of other eIF3 subunits (or the collaboration of subunits within the intact complex) for their translation.

**FIGURE 6:**
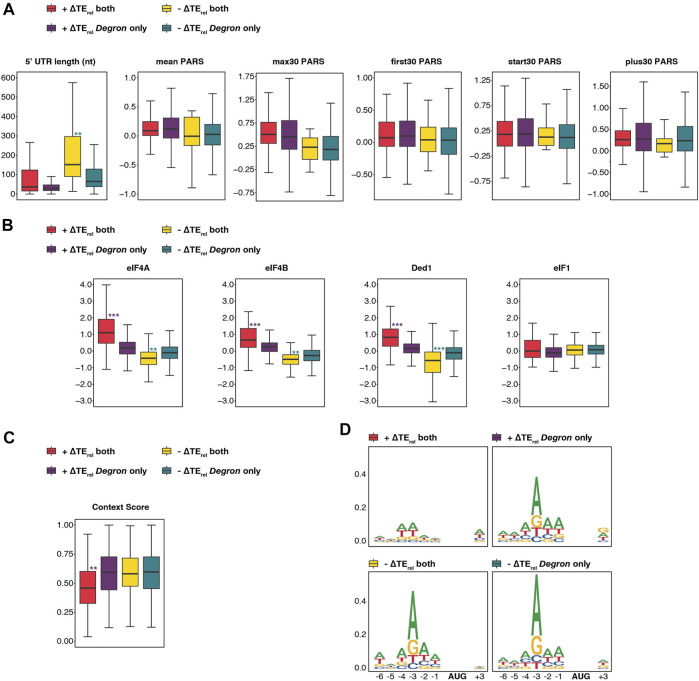
The eIF3 mRNA-entry-channel arm collaborates with eIF4A, eIF4B, and Ded1 to drive initiation on mRNAs with long 5′-UTRs and may also discriminate against poor start-codon context. **(A)** Box and whisker plots comparing different measures of 5′-UTR length or complexity between mRNAs whose TE_rel_ significantly increases or decreases in both *eIF3a/b Degron* and *eIF3i DDKK* cells (red and yellow, respectively) or increases or decreases only in *eIF3a/b Degron* cells (purple and teal, respectively). *** = P_adj_ < 10^−10^, ** = P_adj_ < 10^−5^, * = P_adj_ < 0.05, color indicates comparison set; Wilcoxon Test for 5′-UTR lengths, ANOVA with post-hoc Tukey test for others. **(B)** Same as in A, except comparing ∆TE_rel_ values observed in cells expressing mutations targeting eIF4A, eIF4B, Ded1, and eIF1. *** = P_adj_ < 10^−10^, ** = P_adj_ < 10^−5^, * = P_adj_ < 0.05, color indicates comparison set; ANOVA with post-hoc Tukey test. **(C)** Same as in A, except comparing the Kozak sequence context (nt -6 thru +4) of affected mRNAs. *** = P_adj_ < 10^−10^, ** = P_adj_ < 10^−5^, * = P_adj_ < 0.05. **(D)** Sequence logos of nt -6 to +4 of mRNAs whose TE_rel_ either increases (top panels) or decreases (bottom panels) in both *eIF3a/b Degron* and *eIF3i DDKK* cells (left panels) or only in *eIF3a/b Degron* cells (right panels).

To this end, we compared the features of mRNAs whose TE_rel_ significantly increased or decreased either in both strains or exclusively in *eIF3a/b Degron* cells (Figure 6A). mRNAs whose TE_rel_ decreased in both *eIF3i DDKK* and *eIF3a/b Degron* cells possess longer 5′-UTRs than those whose TE_rel_ decreased only in *eIF3a/b Degron* cells, when restricting our analysis to mRNAs previously identified as having a dominant 5′ transcript isoform (P < 10^−9^). In contrast, there is no significant difference in the 5′-UTR lengths of mRNAs whose TE_rel_ increased. We observed similar results when comparing 5′-UTR lengths reported in a separate study (Kertesz et al., 2010) (Supplementary Figure S5B). We also observed no difference in the degree of structural complexity, as measured by various PARS metrics (Figure 2A), of mRNAs whose TE_rel_ was affected either in both eIF3 mutant backgrounds or solely in *eIF3a/b Degron* cells. These results suggest that the eIF3i and eIF3g subunits of the mRNA-entry-channel arm may be specifically required for the contributions of eIF3 to processive scanning through long 5′-UTRs, whereas the other subunits of the eIF3 complex, either independently or in collaboration with eIF3i and eIF3g, participate in its contribution to the resolution of structural impediments during initial mRNA docking and scanning.

We next compared the differential sensitivity of affected mRNAs within these groups to mutations targeting eIF4A, eIF4B, Ded1, or eIF1 (Sen et al., 2015; Sen et al., 2016; Zhou et al., 2020). mRNAs whose TE_rel_ increased or decreased in both *eIF3i DDKK* and *eIF3a/b Degron* cells were significantly more sensitive to mutations targeting eIF4A, eIF4B, or Ded1 than those uniquely affected in *eIF3a/b Degron* cells: mRNAs whose TE_rel_ decreased in both mutant eIF3 cell lines displayed greater TE_rel_ decreases in response to mutations targeting these factors (P < 10^−9^, P < 10^−8^, and P < 10^−12^ for eIF4A, eIF4B, and Ded1, respectively; ANOVA) and mRNAs whose TE_rel_ increased displayed stronger TE_rel_ increases in these datasets (P < 10^−16^ for all comparisons, Figure 6B). In contrast, we observed no significant differences in the relative sensitivity of affected mRNAs to a mutation targeting eIF1. These differential sensitivities are consistent with a role for the mRNA-entry-channel arm in collaborating with eIF4A, eIF4B, and Ded1 during mRNA recruitment.

Finally, mRNAs whose TE_rel_ increased in both *eIF3i DDKK* and *eIF3a/b Degron* cells possess significantly weaker context scores than transcripts whose TE_rel_ increased uniquely in *eIF3a/b Degron* cells (P < 10^−8^, Figure 6C). Consistent with this, sequence logos reveal that mRNAs whose TE_rel_ increased in both mutant eIF3 cell lines display a weaker preference for adenine at the -3 position (Figure 6D). These observations suggest that eIF3i and eIF3g, and by extension the eIF3 mRNA-entry-channel arm, may play a role in discriminating against AUG codons in poor context.

## Discussion

eIF3 is a multisubunit complex that contributes to events throughout the initiation pathway (Hinnebusch, 2006; Valášek et al., 2017). However, disentangling the contributions of eIF3 and its individual subunits to these events has thus far proved challenging.

To shed light on the mechanistic roles of eIF3 and its component subunits, we interrogated the effects of disrupting either the entire eIF3 complex or the eIF3i and eIF3g subunits—both components of the mRNA-entry-channel arm of eIF3—using ribosome profiling. Our results suggest that the eIF3 complex contributes to driving initiation on mRNAs with long and structurally complex 5′-UTRs and a lower propensity for forming closed-loop structures mediated by eIF4G, eIF4E, and PABP. To a lesser degree, eIF3 may also contribute to discriminating against mRNAs whose start codons appear in weak sequence context. Our results further suggest that eIF3i and eIF3g and thus the eIF3 mRNA-entry-channel arm contribute to the role of eIF3 in facilitating scanning through longer 5′-UTRs, perhaps in collaboration with eIF4A, eIF4B, and Ded1. These subunits may also contribute to discriminating against weak sequence context surrounding the start codon. However, they appear less critical for the role eIF3 plays in resolving structurally complex 5′-UTRs. Instead, the eIF3a, eIF3b, and eIF3c subunits—or all five subunits in collaboration—are required for this role.

Consistent with the strong growth defects provoked by both the *eIF3i DDKK* and *eIF3a/b Degron* mutations (Jivotovskaya et al., 2006; Herrmannová et al., 2012), we observed strong global translational defects in the presence of both mutations. Our ribosome profiling results further identified mRNAs in both mutant backgrounds whose TE_rel_ was significantly affected, as compared to the overall distribution of ∆TE_rel_ values we observed. Whereas the strong global translational effects we observe suggest that the absolute TE of most mRNAs likely decreases in the presence of both mutations, these absolute effects are removed by the normalization of read counts to library size within each sample. Instead, we focus on the relative changes in TE (TE_rel_) we observe in each mRNA, as compared to the overall population of mRNAs across the transcriptome. In the background of global translational suppression that we observe in both cell lines, we interpret these TE_rel_ changes as indicating mRNAs whose translation is more dependent (in the case of negative ∆TE_rel_ values) or less dependent (positive ∆TE_rel_ values) than the overall population of mRNAs.

Despite the strong effects on global translation that we observe in both mutant eIF3 strains, we identify many more mRNAs whose TE_rel_ is significantly affected in *eIF3a/b Degron* cells. Intriguingly, the set of mRNAs most sensitive (−∆TE_rel_) to the *eIF3a/b Degron* mutation was enriched in mRNAs involved in processes such as mitochondrial translation or metabolism. eIF3 was recently implicated in driving the translation of mitochondrial mRNAs in both fission yeast (Shah et al., 2016) and mammalian cells (Lin et al., 2020), a role which was attributed to the eIF3e and eIF3d subunits. eIF3d has also been linked to the preferential translation of mRNAs involved in cell proliferation pathways in human cells (Lee et al., 2015; Lee et al., 2016). This latter regulatory role appears to involve a cap-independent initiation mechanism driven by eIF3. This emerging regulatory role for eIF3 may also be linked to the observation that eIF3 binding to the PIC persists through early rounds of elongation in both yeast and mammalian systems (Bohlen et al., 2020; Lin et al., 2020; Wagner et al., 2020). However, neither eIF3d nor eIF3e is present in the budding yeast complex. Our results suggest that the five subunits of the yeast core complex may nonetheless be capable of driving the selective translation of specific mRNAs. More targeted disruption of these subunits might further illuminate the origin of these effects and whether they involve the participation of eIF3 during initiation or early elongation cycles.

In contrast, we did not observe significant enrichment of specific GO terms in the sets of transcripts whose TE_rel_ was affected in *eIF3i DDKK* cells. We further observed a narrower set of transcripts whose translation is affected either more or less strongly than the overall population of mRNAs in the presence of this specific disruption of the eIF3 mRNA-entry-channel arm. That these cells still display strong defects in global translation—and thus likely decreases in the absolute TE of most mRNAs—suggests that the eIF3i and eIF3g subunits might contribute to aspects of mRNA recruitment required more universally across the transcriptome, as opposed to being required to drive translation of specific classes of mRNAs. This was recently suggested for eIF4A in light of the observation that ribosome profiling of cells expressing a temperature-sensitive eIF4A variant that provokes strong global translational defects did not identify substantial numbers of mRNAs more or less sensitive than the overall population, consistent with *in vitro* measurements suggesting a universal role for eIF4A in alleviating structural complexity within mRNAs (Sen et al., 2015; Yourik et al., 2017).

While our results point to potentially distinct roles for eIF3 and its mRNA-entry-channel arm in either mediating initiation on specific classes of mRNAs or driving the translation of mRNAs across the transcriptome, they also further illuminate the role of eIF3 and its mRNA-entry-channel arm in contributing to mRNA recruitment and its component events of PIC docking, scanning, and start-codon recognition. We show that mRNAs possessing longer 5′-UTRs are more sensitive to disruption of both the entire complex or targeted disruption of the mRNA-entry-channel arm. This is consistent with the identification of mutations throughout the eIF3 complex that affect scanning, with several of these mutations targeting eIF3i (Herrmannová et al., 2012), eIF3g (Cuchalova et al., 2010), and other constituents of the mRNA-entry-channel arm (Nielsen et al., 2006; Chiu et al., 2010; Cuchalova et al., 2010).

We also observed that the effects of both eIF3 mutations are similar to those observed via ribosome profiling of cells expressing mutations targeting eIF4A, eIF4B, and Ded1 (Sen et al., 2015; Sen et al., 2016; Zhou et al., 2020), all of which are thought to contribute either to initial PIC docking to the mRNA or subsequent scanning. These similarities extend to the observation that mRNAs likely to form stable closed-loop structures are least sensitive to mutations targeting these initiation factors and to both eIF3 mutations, whereas mRNAs less likely to form stable closed-loop structures are more sensitive. Together, these observations suggest that eIF3 may collaborate with eIF4A, eIF4B, and Ded1 to drive initiation on mRNAs with longer 5′-UTRs and a weaker dependence on the initiation factors eIF4G, eIF4E, and PABP. eIF3—via the eIF3a CTD component of the eIF3 mEnC arm—interacts with eIF4B (Methot et al., 1996) and with the 40S latch (Chiu et al., 2010). Moreover, eIF3 is present in both the mRNA-entry and -exit channels of the ribosome in both yeast and mammalian structures (Aylett et al., 2015; Simonetti et al., 2016). In a recent structure of the human 48S complex, eIF3g was observed binding to ribosomal RNA and protein elements within the mRNA-entry channel and eIF3k, eIF3l, and eIF3e were found adjacent to eIF4A near the mRNA-exit channel (Querido et al., 2020). And yet, eIF3k, -l, and -e are absent from the yeast eIF3 complex, where eIF3a and eIF3c are alone found near the mRNA-exit channel of the ribosome. However, *in vitro* studies reveal that, in addition to eIF3d and eIF3e, eIF3c (which is present in budding yeast) is also able to bind to components of the eIF4F complex (Villa et al., 2013). Together with our results here, these observations together raise several possibilities for direct or functional collaboration between eIF3 and these other factors.

eIF3 has also been implicated in driving cap-independent initiation mechanisms (Lee et al., 2016; Rode et al., 2018; Bhardwaj et al., 2019) and in directly recruiting the PIC to specific classes of mRNAs (Lee et al., 2015; Lee et al., 2016; Shah et al., 2016; Lin et al., 2020; Lamper et al., 2020), both of which may circumvent the requirement for eIF4G-, eIF4E-, and PABP-mediated closed-loop formation. Our observation that these effects manifest upon targeting either the entire eIF3 complex or simply the mRNA-entry-channel arm point to a role for the mRNA-entry-channel arm in collaborating with these other factors to mediate initiation in the absence of stable closed-loop formation. In fact, eIF3 and eIF4A were recently shown to collaborate in a non-canonical initiation pathway that circumvents eIF4E and eIF4G during neuronal development in *Drosophila* (Rode et al., 2018).

In contrast, we observed stronger effects of mRNA structural complexity when disrupting the entire eIF3 complex than when specifically targeting its mRNA-entry-channel arm. Certainly, this does not exclude the possibility that either or both eIF3i and eIF3g contribute to resolving regions of structural complexity within the 5′-UTR. Mutations targeting these subunits have previously been shown to interfere with initiation on reporter mRNAs containing stable stem loop structures (Cuchalova et al., 2010; Herrmannová et al., 2012). Nonetheless, our results point to the remaining eIF3 subunits—eIF3a, eIF3b, and eIF3c, either alone or in collaboration with eIF3i and eIF3g—playing a role in resolving structural complexity during initial PIC docking or scanning. Both eIF3a (via its CTD) and eIF3b contribute to the mRNA-entry-channel arm (Aylett et al., 2015; Llácer et al., 2015; Simonetti et al., 2016; Llácer et al., 2018) and mutations targeting the eIF3a CTD disrupt initiation on reporter mRNAs containing stem loop structures (Chiu et al., 2010). In addition, eIF3a interacts physically and functionally with mRNA near the mRNA-exit channel of the ribosome (Szamecz et al., 2008; Munzarová et al., 2011; Aitken et al., 2016; Llácer et al., 2018) and eIF3c binds components of the eIF4F complex (Villa et al., 2013) that have recently been visualized near the mRNA-exit channel in the human 48S PIC (Querido et al., 2020).

Whereas we observed relatively strong effects on either initial docking or scanning in the presence of both eIF3 mutations, we observed more nuanced effects on start-codon recognition upon disruption of the eIF3 mRNA-entry-channel arm. In contrast to the similarities between the effects of both eIF3 mutations and those previously observed for mutations targeting eIF4A, eIF4B, and Ded1, we observed relatively little similarity with those observed in previous ribosome profiling experiments targeting eIF1. Nonetheless, our observation that those mRNAs least sensitive to the *eIF3i DDKK* mutation exhibit weaker start-codon context does suggest that the eIF3 mRNA-entry-channel arm plays a role in discriminating against AUG codons in weak context. Consistent with this, mutations targeting several components of the mRNA-entry-channel arm elicit defects in either the accuracy or efficiency of start-codon recognition (Nielsen et al., 2004, 2006; Chiu et al., 2010; Cuchalova et al., 2010; Herrmannová et al., 2012). The fact that we observe these modest effects but do not observe similarities between our dataset and ribosome profiling data from eIF1 mutant cells suggests that these effects do not arise from disruption of the functional collaboration between eIF3 and eIF1(Valasek et al., 2004; Llácer et al., 2015; Llácer et al., 2018). Instead, it is possible that disruption of the eIF3 mRNA-entry-channel arm might disrupt its modulation of the equilibrium between the open and closed states of the PIC via interaction with the 40S latch. Indeed, mutations targeting this nexus produce start-codon recognition defects (Chiu et al., 2010; Dong et al., 2017).

Overall, we observed striking similarities between the effects of the *eIF3i DDKK* and *eIF3a/b Degron* mutations. Consistent with their disruption of either a portion of the eIF3 mRNA-entry-channel arm (*eIF3i DDKK*) or the entire complex (*eIF3a/b Degron*), the affected mRNAs we identified in *eIF3i DDKK* cells comprise subsets of those we identified in *eIF3a/b Degron* cells. Nonetheless, comparison of those transcripts whose TE_rel_ was affected in both eIF3 mutant backgrounds with those affected solely in *eIF3a/b Degron* cells identifies telling differences that suggest roles for the eIF3i and eIF3g subunits of the eIF3 mRNA-entry-channel arm. mRNAs whose TE_rel_ decreases in both backgrounds—suggesting that their translation depends more strongly on the eIF3i and eIF3g subunits affected by both mutations—have 5′-UTRs that are longer but are no more structurally complex than those whose TE_rel_ decreases only when the entire eIF3 complex is disrupted. mRNAs sensitive to both mutations are also more strongly affected by mutations targeting eIF4A, eIF4B, and Ded1. mRNAs whose TE_rel_ decreased in both backgrounds displayed stronger TE_rel_ decreases in the presence of these other mutations than mRNAs whose TE_rel_ decreased solely in *eIF3a/b Degron* cells. mRNAs whose TE_rel_ increased in both mutant eIF3 backgrounds similarly display stronger TE_rel_ increases in cells expressing mutants of eIF4A, eIF4B, or Ded1. mRNAs whose TE_rel_ increased in both eIF3 backgrounds further possess weaker start-codon sequence context than those whose TE_rel_ increased solely in *eIF3a/b Degron* cells.

Together, these observations again point to roles for eIF3i and eIF3g, and by extension the eIF3 mRNA-entry-channel arm, in functional collaboration with eIF4A, eIF4B, and Ded1 to drive processive scanning through longer 5′-UTRs on mRNAs whose translation is less dependent on the formation of a stable closed loop. Surprisingly, effects on scanning processivity were not previously observed using a set of reporter constructs in extracts derived from cells expressing *eIF3i DDKK* cells (Herrmannová et al., 2012), perhaps because other features present in natural mRNAs or mRNPs are required to elicit these effects. Distinct mutations targeting either eIF3i or eIF3g, however, do manifest defects in scanning processivity (Cuchalova et al., 2010). Our results also suggest that eIF3i and eIF3g play a role in discriminating against weak start-codon context during start-codon recognition. Previous experiments following the effects of the *eIF3i DDKK* mutation using a series of reporter mRNAs observed leaky scanning of AUG codons, suggesting these subunits might contribute to efficient start-codon recognition (Herrmannová et al., 2012). However, the effect of codon context was not reported.

While the eIF3i and eIF3g subunits of the eIF3 mRNA-entry-channel arm appear to contribute to scanning processivity, the remaining subunits (or the entire complex) appear to contribute to resolving structural complexity within 5′-UTRs. We observed stronger and more significant effects of various measures of structural complexity throughout the 5′-UTRs of mRNAs in *eIF3a/b Degron* cells than we did in *eIF3i DDKK* cells. That we do not observe these effects in *eIF3i DDKK* cells but still observe strong similarities with those effects observed in mutant eIF4A, eIF4B, or Ded1 cells (and find that mRNAs sensitive only to the *eIF3a/b Degron* mutation are less sensitive to mutations targeting these factors) might suggest that eIF3 can also contribute to the resolution of structural complexity independently of any collaboration with these factors. Another possibility is that eIF3 does indeed collaborate with these factors but via distinct functional mechanisms to resolve stable structural impediments near the 5′ cap or within downstream 5′-UTR regions.

Finally, we also found that mRNAs whose translation is most sensitive to the disruption of the entire eIF3 complex were enriched in mRNAs involved in mitochondrial translation and metabolism. This observation echoes the recently identified role of eIF3 in preferentially mediating translation on these classes of mRNAs in fission yeast and mammalian cells (Shah et al., 2016; Lin et al., 2020). That role appears to involve the eIF3d subunit, which has also been implicated in mediating the translation of mRNAs involved in cell proliferation (Lee et al., 2016), as well as the ability of eIF3 to remain bound during early rounds of elongation (Bohlen et al., 2020; Lin et al., 2020; Wagner et al., 2020). Because eIF3d is absent in budding yeast cells, our observations suggest that at least one subunit of the core complex is capable of reprising aspects of this role. A potential candidate is eIF3a which, like eIF3d, is positioned near the mRNA-exit channel of the ribosome and appears to interact physically and functionally with the mRNA there (Szamecz et al., 2008; Aitken et al., 2016; Llácer et al., 2018). eIF3a has also previously been implicated in mediating sequence-dependent reinitiation events, a function which requires direct interaction with specific mRNA sequence elements (Szamecz et al., 2008; Munzarová et al., 2011). The mechanistic origin of these effects in budding yeast, and whether they involve the participation of eIF3 during initiation, early rounds of elongation, or both, emerge as intriguing questions.

Our work sheds light on the specific roles of the eIF3 mRNA-entry-channel arm and its other subunits during the component events of mRNA recruitment. It further points to a potential role for eIF3 in mediating the translation of specific classes of mRNAs, as in higher eukaryotic cells. Nonetheless, experiments following the fate of reporter constructs containing the 5′-UTRs of sensitive mRNAs in *eIF3i DDKK* and *eIF3a/b Degron* cells or cell extracts or the requirements of sensitive mRNAs for eIF3 or eIF3i and eIF3g in mRNA recruitment assays *in vitro* might further strengthen the case for these roles. Still further investigation is necessary to determine the mechanism whereby eIF3 mediates translation of these mRNAs, how it functions to facilitate initiation on mRNAs with structurally complex 5′-UTRs, and how its mRNA-entry-channel arm collaborates with other initiation factors to drive initial docking and scanning on mRNAs independent of closed-loop formation.

## Data Availability

Sequencing data from this study have been submitted to the NCBI Gene Expression Omnibus (GEO; http://www.ncbi.nlm.nih.gov/geo/) under accession number GSE190601.

## Supplementary Material

Supplementary Figure 1 Disruption of the eIF3 complex or its mRNA-entry-channel arm provoke strong global translational defects. **(A)** Polysome profiles collected from *eIF3a/b Degron* and *eIF3i DDKK* cells and isogenic wild-type cells grown under permissive conditions. **(B,C)** Polysome profiles collected from biological replicates used to prepare ribosome profiling libraries for *eIF3i DDKK* **(B)** and *eIF3a/b Degron* (**C**) cells and isogenic wild-type cells upon shifting to growth under restrictive conditions for 30 min (*eIF3i DDKK*) or 90 min (*eIF3a/b Degron*).

Supplementary Figure 2 mRNA fragment and Ribosome footprint libraries obtained from biological replicates of *eIF3i DDKK* and *eIF3a/b Degron* cells are highly reproducible. Correlation plots of read counts obtained from mRNA fragment and ribosome footprint libraries prepared from biological replicates of *eIF3i DDKK* and *eIF3a/b Degron* cells and matching isogenic wild-type cells.

Supplementary Figure 3 Disruption of the eIF3 complex affects the translation of mRNAs associated with mitochondrial processes. Bar plots showing the percent over- or under-representation of specific biological process GO terms in the list of mRNAs whose TE_rel_ either significantly decreases (left) or increases (right) in *eIF3a/b Degron* cells. Color scale indicates P_adj_ values.

Supplementary Figure 4 Disruption of eIF3 or its mRNA-entry-channel arm result in global increases in relative uORF translation that are strongest for uORFs closest to the 5’ end. **(A)** Correlation of uORF TE_rel_ values observed in either *eIF3a/b Degron* (left) or *eIF3i DDKK* (right) cells and those observed in isogenic wild-type cells. uORFs displaying significant (P_adj_ < 0.05) TE_rel_ increases (>50% red, > 100% blue) or decreases (>50% yellow, >100% green) as determined by DESeq2 analysis are shown in colors. **(B)** Correlation of observed ∆TE_rel_ values and distance from the 5’ end for individual uORFs in *eIF3a/b Degron* (left) and *eIF3i DDKK* (right) cells. Spearman correlation fits shown in red.

Supplementary Figure 5 Disruption of eIF3 or its mRNA-entry-channel arm affect the translation of mRNAs with long 5’-UTRs. **(A)** Box and whisker plots comparing 5’-UTR lengths (reported by Kertesz, et al., Nature 2010) between mRNAs whose TE_rel_ significantly increases (red), decreases (yellow), or is unaffected (white) in each eIF3 mutant strain. *** = P_adj_ < 10^-10^, ** = P_adj_ < 10^-5^, * = P_adj_ < 0.05, color indicates comparison set; Wilcoxon test. **(B)** Same as in A, except comparing 5’-UTR lengths between mRNAs whose TE_rel_ significantly increases or decreases in both *eIF3a/b Degron* and *eIF3i DDKK* cells (red and yellow, respectively) or increases or decreases only in *eIF3a/b Degron* cells (purple and teal, respectively). *** = P_adj_ < 10^-10^, ** = P_adj_ < 10^-5^, * = P_adj_ < 0.05, color indicates comparison set; Wilcoxon test.

Supplementary Figure 6 Observed ∆TE_rel_ values in *eIF3a/b Degron* and *eIF3i DDKK* cells are not correlated with the AUG sequence context of individual mRNAs. Correlation plots comparing observed ∆TE_rel_ values in *eIF3a/b Degron* (left) or *eIF3i DDKK* (right) cells and the AUG sequence context (nt −6 to +4) of individual mRNAs. Predicted Spearman correlations shown in red.

Supplementary Figure 7 ∆TE_rel_ values observed in *eIF3a/b Degron* and *eIF3i DDKK* cells strongly correlate with those observed in the presence of mutations targeting eIF4A, eIF4B, and Ded1 but not eIF1 or eIF1A. Correlation plots comparing observed ∆TE_rel_ values in *eIF3a/b Degron* (top) or *eIF3i DDKK* (bottom) cells and those observed for mutations targeting eIF4A, eIF4B, Ded1, eIF1, and eIF1A. Predicted Spearman correlations shown in red.
