# Corrigendum: eIF3 and Its mRNA-Entry-Channel Arm Contribute to the Recruitment of mRNAs With Long 5′-Untranslated Regions

**DOI:** 10.3389/fmolb.2022.896119

**Published:** 2022-04-20

**Authors:** Andrei Stanciu, Juncheng Luo, Lucy Funes, Shanya Galbokke Hewage, Shardul D. Kulkarni, Colin Echeverría Aitken

**Affiliations:** ^1^ Computer Science Department, Vassar College, Poughkeepsie, NY, United States; ^2^ Biochemistry Program, Vassar College, Poughkeepsie, NY, United States; ^3^ Biology Department, Vassar College, Poughkeepsie, NY, United States; ^4^ Department of Biochemistry & Molecular Biology, Penn State Eberly College of Medicine, University Park, PA, United States

**Keywords:** eIF3, translation initiation, translational regulation, mRNA recruitment, ribosome, ribosome profiling, Ribo-seq

In the published article, **Shardul D. Kulkarni** was not included as an author. Author list has been corrected and the Author contributions section has been updated as appears below.

“CA performed the experiments, wrote the manuscript, prepared final versions of the figures, and performed data analysis. AS performed significant data preparation, analysis, and interpretation. JL performed data analysis and interpretation. LF and SG performed data analysis. SK performed sample preparation and data preparation.”

The authors apologize for this error and state that this does not change the scientific conclusions of the article in any way. The original article has been updated.

