# Supplementary figures and images for: eIF3 and Its mRNA-Entry-Channel Arm Contribute to the Recruitment of mRNAs With Long 5′-Untranslated Regions
Source: Front Mol Biosci. 2022 Jan 11;8:787664. doi: 10.3389/fmolb.2021.787664 (PMC8787345; doi:10.3389/fmolb.2021.787664)

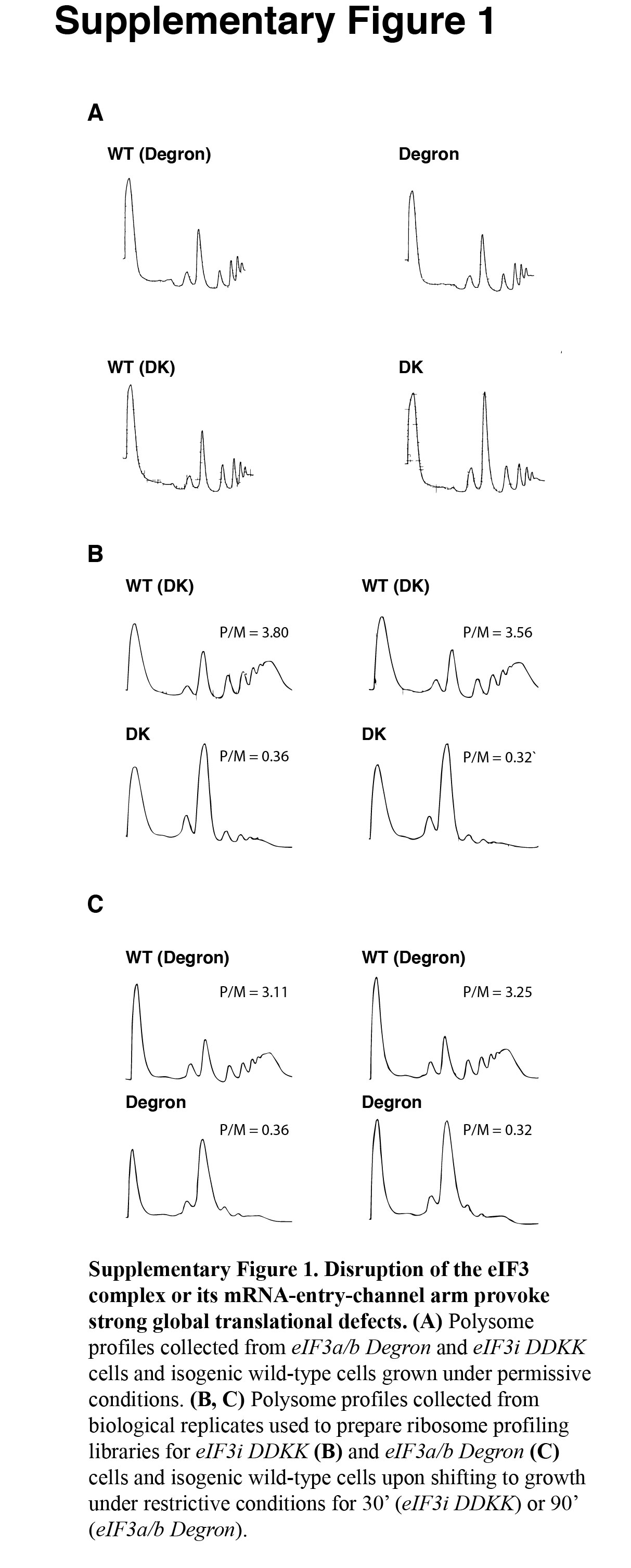

Supplement: Supplementary file 1 [file Presentation1.zip › Figure 1.JPEG]

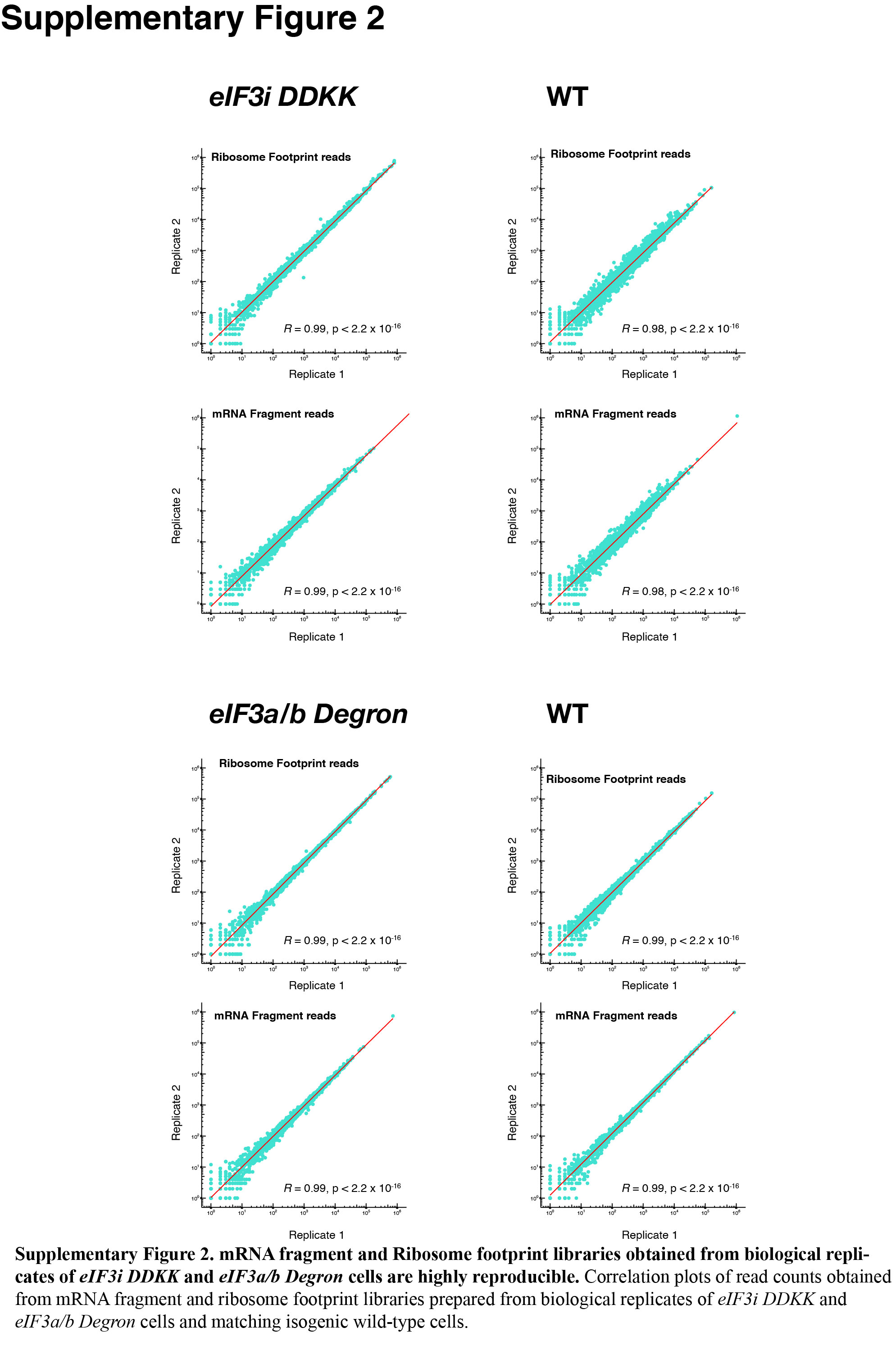

Supplement: Supplementary file 1 [file Presentation1.zip › Figure 2.JPEG]

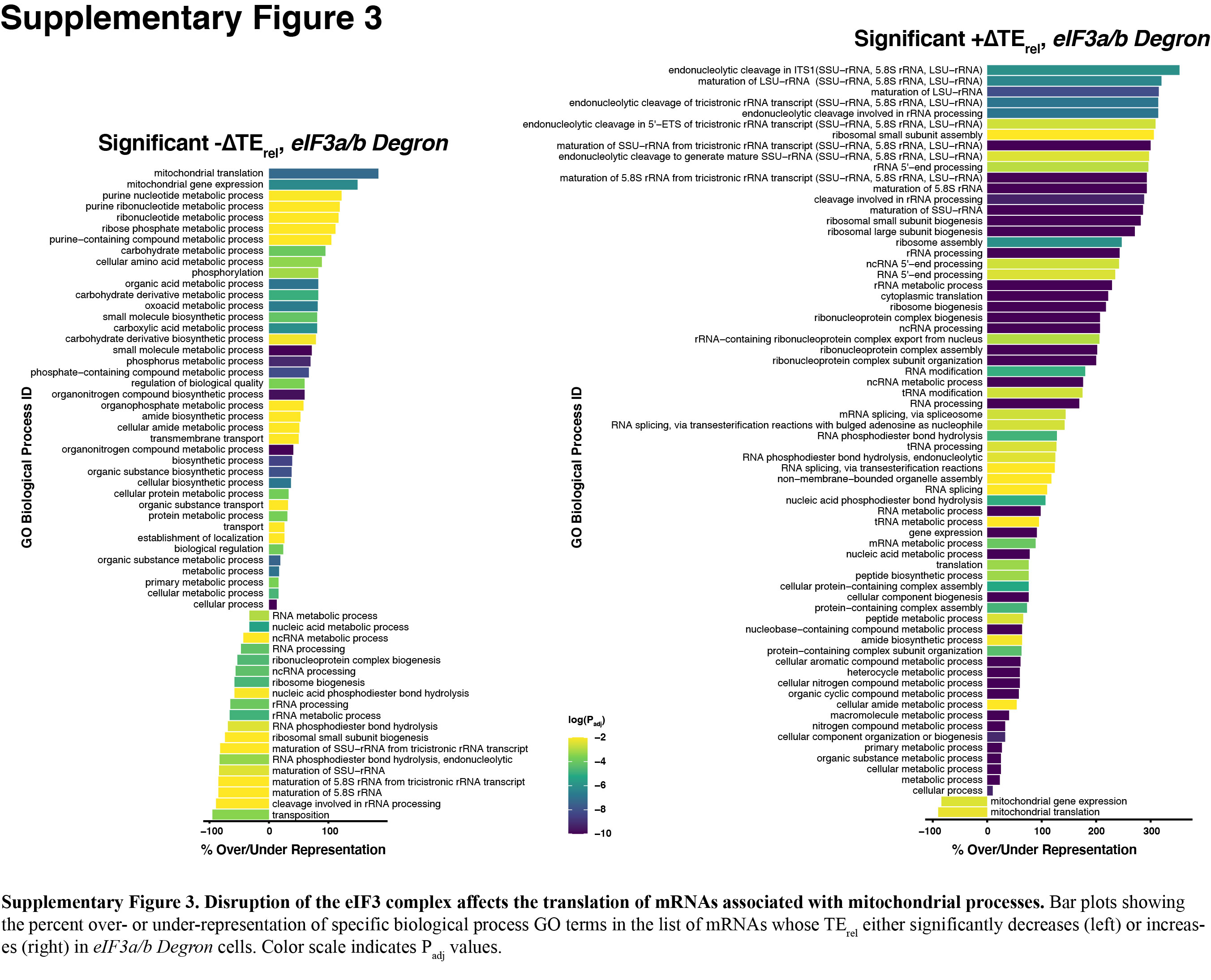

Supplement: Supplementary file 1 [file Presentation1.zip › Figure 3.JPEG]

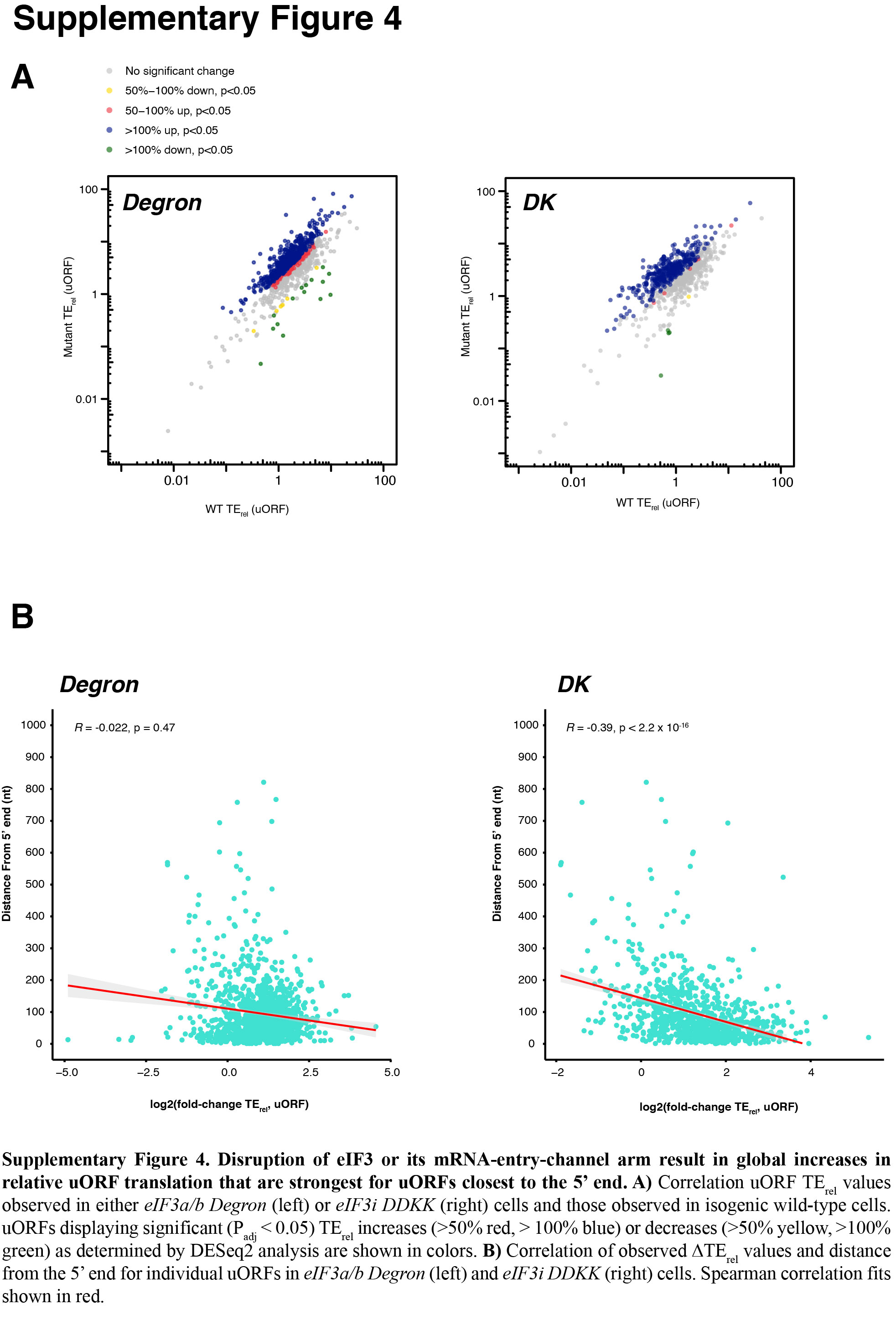

Supplement: Supplementary file 1 [file Presentation1.zip › Figure 4.JPEG]

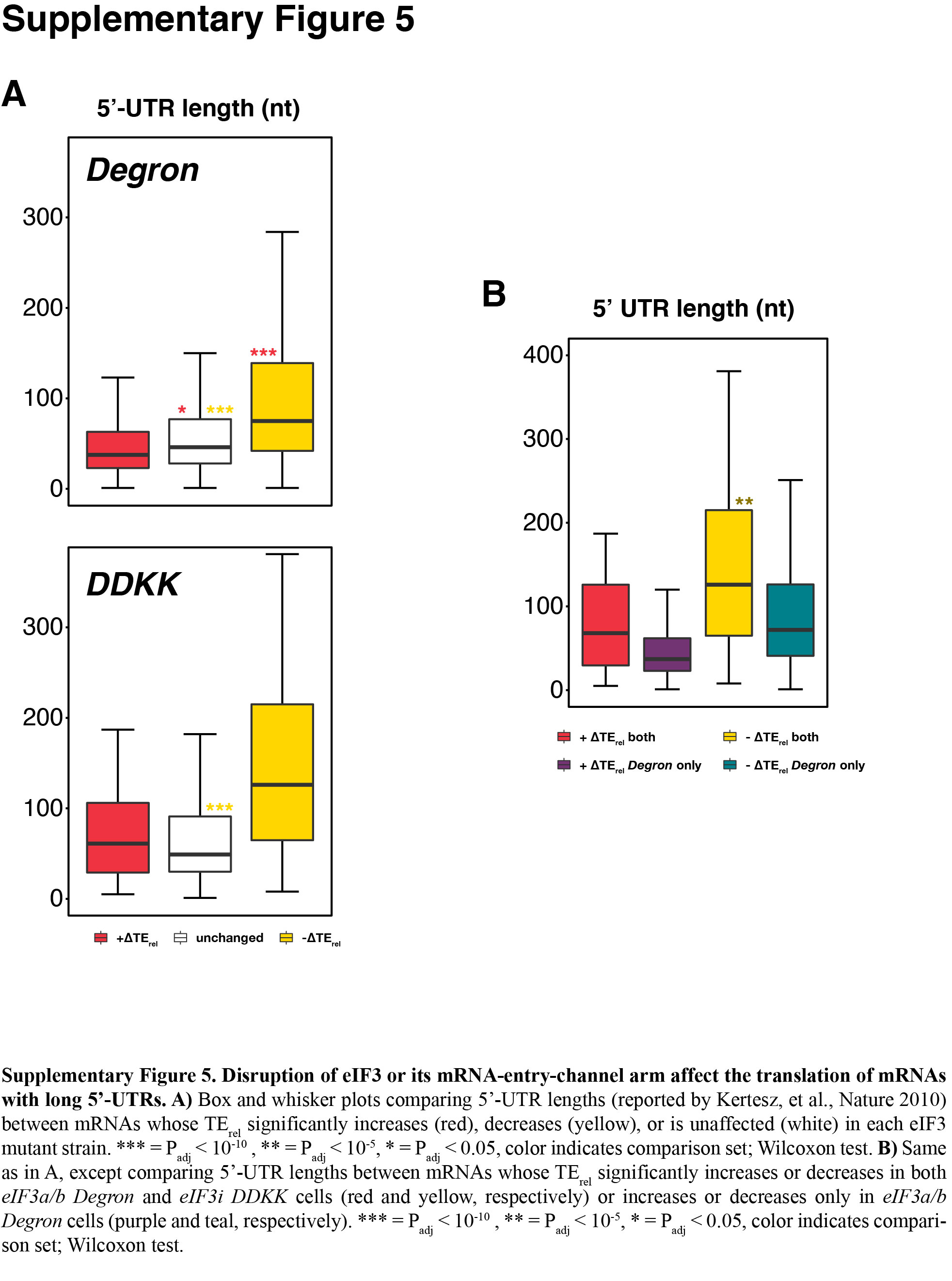

Supplement: Supplementary file 1 [file Presentation1.zip › Figure 5.JPEG]

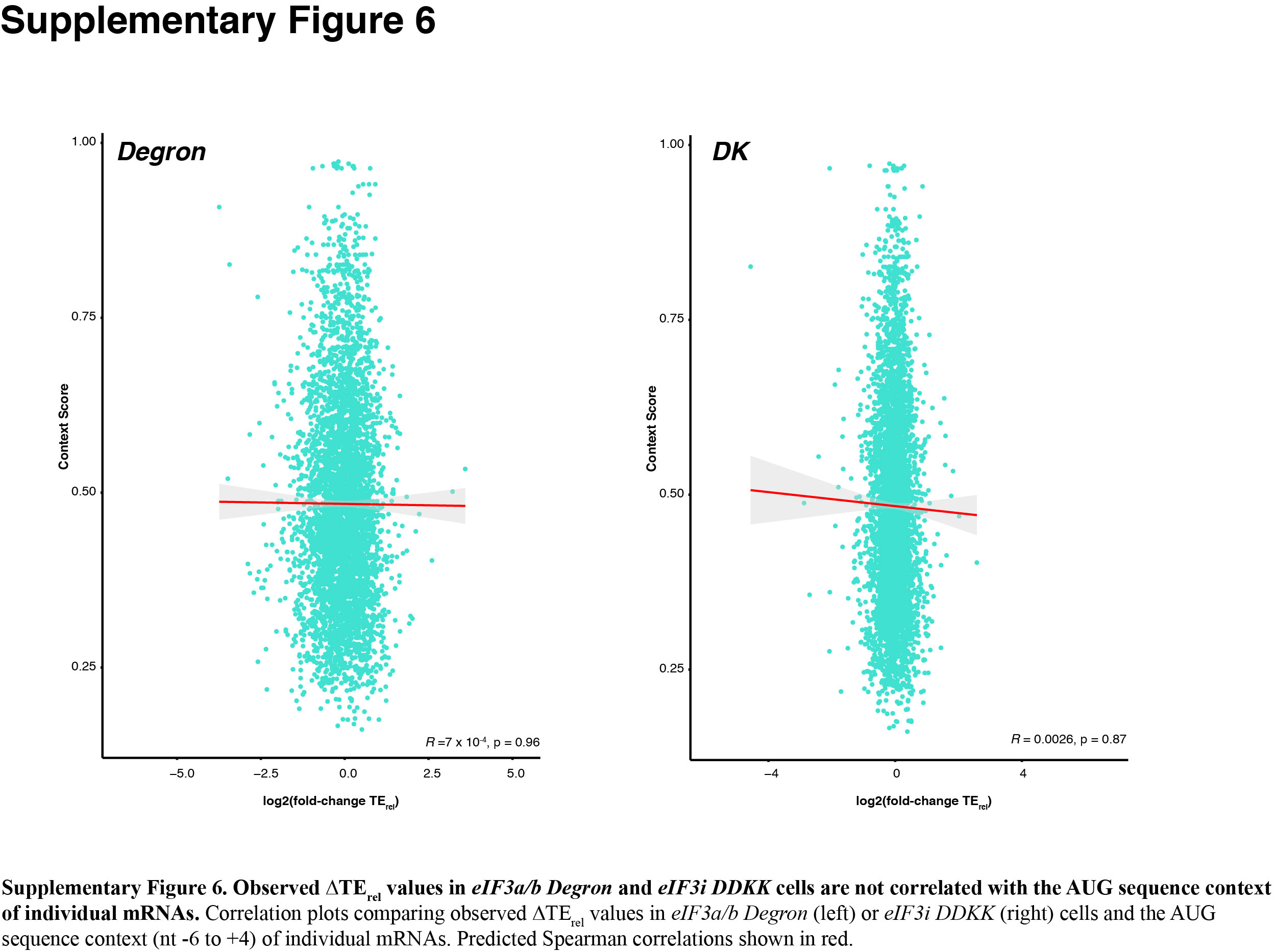

Supplement: Supplementary file 1 [file Presentation1.zip › Figure 6.JPEG]

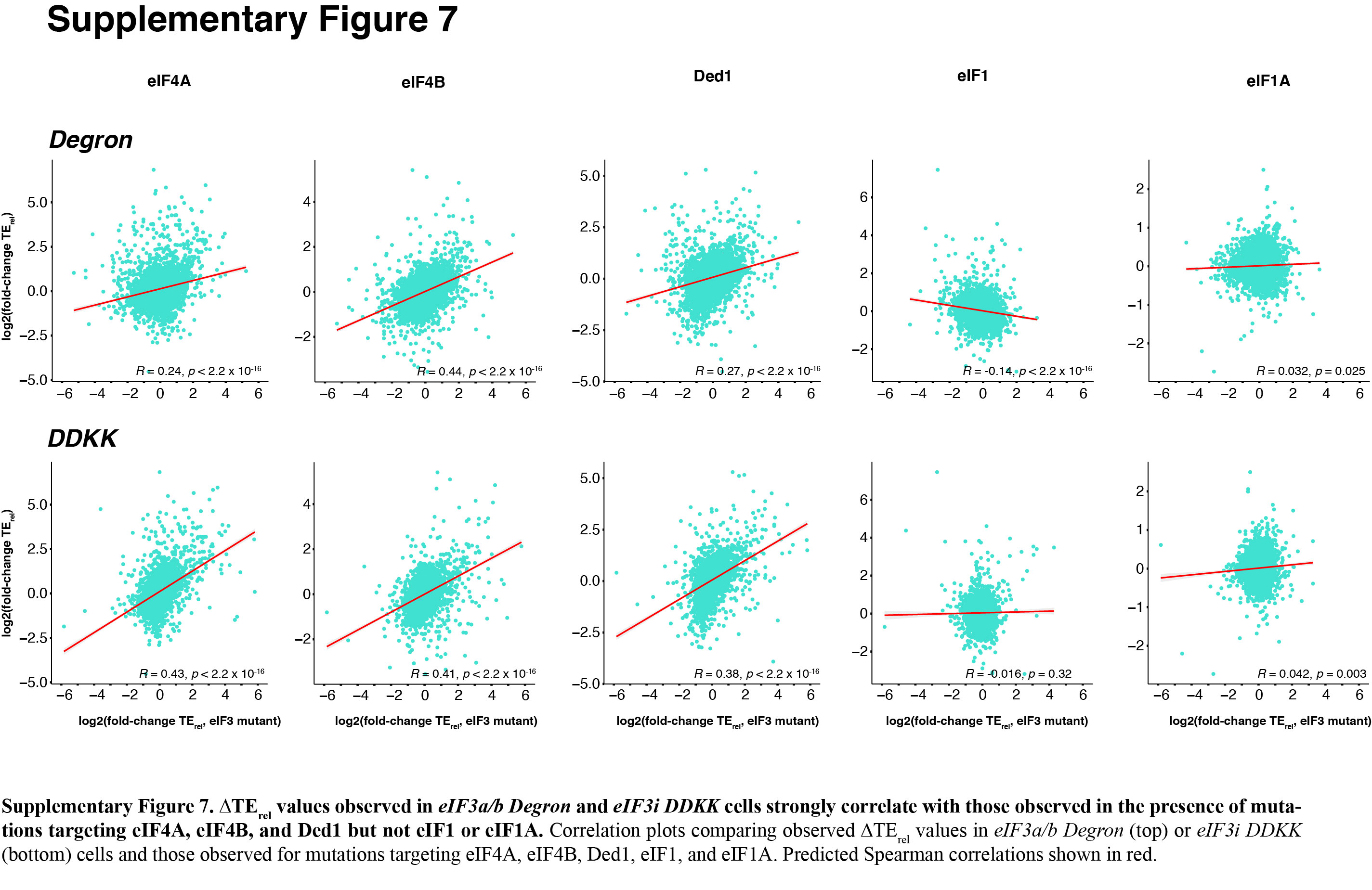

Supplement: Supplementary file 1 [file Presentation1.zip › Figure 7.JPEG]
